# Crotonylation impedes c-Myc oncogenic activity

**DOI:** 10.1073/pnas.2530020123

**Published:** 2026-06-01

**Authors:** Nicholas J. Wallbillich, Peng Liao, Rashmi Srivastava, Jia Fan, Shelya X. Zeng, Hua Lu

**Affiliations:** ^a^ Department of Biochemistry and Molecular Biology, Tulane University School of Medicine, 1430 Tulane Avenue, New Orleans, LA 70112; ^b^Tulane Cancer Center, 1700 Tulane Ave, New Orleans, LA 70112

**Keywords:** c-Myc, posttranslational modification, crotonylation, Skp2, oncogenesis

## Abstract

c-Myc, an important oncoprotein, is highly regulated via posttranslational modifications. Herein, we report c-Myc crotonylation, an acylation stemming from the short-chain fatty acid crotonate. By biochemical analyses and high-resolution mass spectrometry sequencing, we showed that c-Myc is crotonylated at several lysine residues, spanning its middle to C termini. Mutation of these crotonylation sites conferred cells with a significant proliferative advantage with two key residues at K289 and K298 identified. Mutation of these lysine residues increased the binding of the mutant c-Myc to its regulator S-phase-kinase-associated protein 2 (Skp2) that can enhance c-Myc transcriptional activity while degrading it afterward. Interestingly, the K298N mutation was identified in some primary human tumors via screening human cancer database. More interestingly, this cancer-derived mutant c-Myc displayed more oncogenic activity than did wild type c-Myc in vitro and in vivo, in part, by partnering with Skp2. Together, these results demonstrate that crotonylation can impair the oncogenic activity of c-Myc.

## Significance

Only a handful of posttranslational modifications have been reported for c-Myc, one of the key oncoproteins which is overexpressed in an estimated 70% of cancers and responsible for regulating many essential facets of tumorigenesis and therapy resistance. Our study provides evidence for c-Myc crotonylation and identifies two key lysine residues which are responsible for crotonylation induced loss of c-Myc transcriptional activity. A unique mechanism was elucidated in which crotonylation impairs binding of c-Myc to one of its critical positive regulators and ubiquitin ligases, Skp2. Last, a cancer-derived c-Myc mutant sharing one of the key crotonylation residues was identified in human primary tumors, and it displayed higher oncogenic activity in-vitro and in-vivo, in part, by increased binding to Skp2.

Cancer is a heterogenous disease generally characterized by the activation of oncoproteins, such as c-Myc, and the downregulation of tumor suppressors (1, 2). c-Myc is a master transcriptional factor that regulates the expression of more than 15% of mammalian genes involved in cell division, metabolism, ribosomal biogenesis, protein translation, apoptosis, DNA repair, migration, immune response, and stem cell formation (3). c-Myc is highly expressed in over 70% of human cancers, including colon, lung, breast, lymphoma, prostate, and leukemia, particularly in aggressive forms of cancer (4–6). As one of the most significant oncoproteins, it has become a major target for therapeutic development, despite difficulties in its pharmaceutical targeting, namely its intrinsically disordered structure and potential for off-target effects in noncancerous cells (3). Its dysregulation occurs at multiple nodes: DNA amplification, transcription, mRNA stability, protein translation, and, significantly herein, posttranslational modifications (PTMs) (7–10). c-Myc is subject to several PTMs that affect its stability, activity, and subcellular location (9). Phosphorylation and ubiquitination are well studied PTMs that are crucial in determining c-Myc activity level in the cell (11). Phosphorylation by several kinases, including ERK, CDKs, JNK, and GSK3β, on the N terminus, specifically at T58 and S62, are critical in regulation of the stability and activity of c-Myc, with the former promoting its ubiquitin-proteasomal degradation, and the latter promoting its stability and increased transcriptional activity (12). Ubiquitination by a number of E3 ligases has been shown to affect the stability, activity, or both, of c-Myc (13). Notably, the SCF ubiquitin ligase containing the F-box substrate containing protein, Fbw7, was found to ubiquitinate the transactivation domain (TAD), dependent on T58 phosphorylation, and lead to decreased stability and activity (14). Fbw7 is categorized as a tumor suppressor and is mutated in a number of different epithelial cancers (15). Also, mutations near or at T58 in the c-Myc alleles in Burkitt’s lymphoma have been identified (16). The other important ubiquitin ligase is the SCF containing the S-phase kinase associated protein 2 (Skp2) (17). It was the first discovered ubiquitin ligase for c-Myc, originally identified in yeast as essential for c-Myc proteolysis (18). In contrast to Fbw7, Skp2 can stimulate c-Myc transcriptional activity (19). Strikingly, this stimulation was coupled with Skp2 ubiquitination and subsequent proteolysis of c-Myc, suggesting Skp2 as an oncoprotein (20). Consistently, Skp2 levels are elevated in a number of cancers (21).

Acetylation of c-Myc occurs on lysine residues by a number of histone acetyl-transferases (HATs), such as p300/CBP, TIP60, and mGCN5 (22, 23). A connection between ubiquitination and acetylation, due to the overlap of acetylation sites and ubiquitination sites, as well as some c-Myc acetylation defective mutants showing differences with the wild type in ubiquitination assays, has always been deemed likely (24, 25). Acetylation and ubiquitination in other proteins, such as p53, have been shown to be competitive on lysine residues (26, 27). However, not until recently, has acetylation been suggested to be involved in the recruitment of ubiquitin ligases or deubiquitinases, as has been demonstrated for an emerging list of proteins (28, 29). Most recently, acetylation at K148 of c-Myc was shown to promote the binding of c-Myc to USP10, decreasing its ubiquitination and increasing its stability and activity (29).

Crotonylation is a novel form of acylation initially identified as a histone modification on lysine residues, with evolutionary conservation from humans to lower species such as *Drosophila* (30). It originates from the molecule crotonate that is a short-chain fatty acid as well as a metabolic intermediate. Studies with pan-crotonylation antibodies and high-resolution mass spectrometry have extended crotonylation targets to many nonhistone proteins as well (31). While studying p53 crotonylation (32) we also found c-Myc crotonylation, which was coincident with a global proteomic screening (31). In our attempt to understand the role of crotonylation in c-Myc regulation, we validated c-Myc as a crotonylation target using a combination of techniques, including coimmunoprecipitation (IP)-Western blotting (WB) with pan anti-crotonylation antibodies, high-resolution mass spectrometry (HRMS), and chemical in-vitro assay (33). Interestingly, crotonylation-deficient mutants showed an increased activity with increased ubiquitination, which was found to be mediated by the Skp2 ubiquitin ligase. More interestingly, we identified cancer-derived mutant c-Myc at K298N by screening a human cancer database (34, 35) which is one of the two critical crotonylation target residues. Our further studies of this cancer-derived mutant K298N using cell-based assays and a xenograft tumor model system as detailed below demonstrate that this crotonylation-deficient mutant c-Myc is more active than its wild type counterpart in promoting colorectal cancer cell growth in vitro and in vivo.

## Results

### c-Myc Is Crotonylated in Human Cells.

**Fig. 1.**
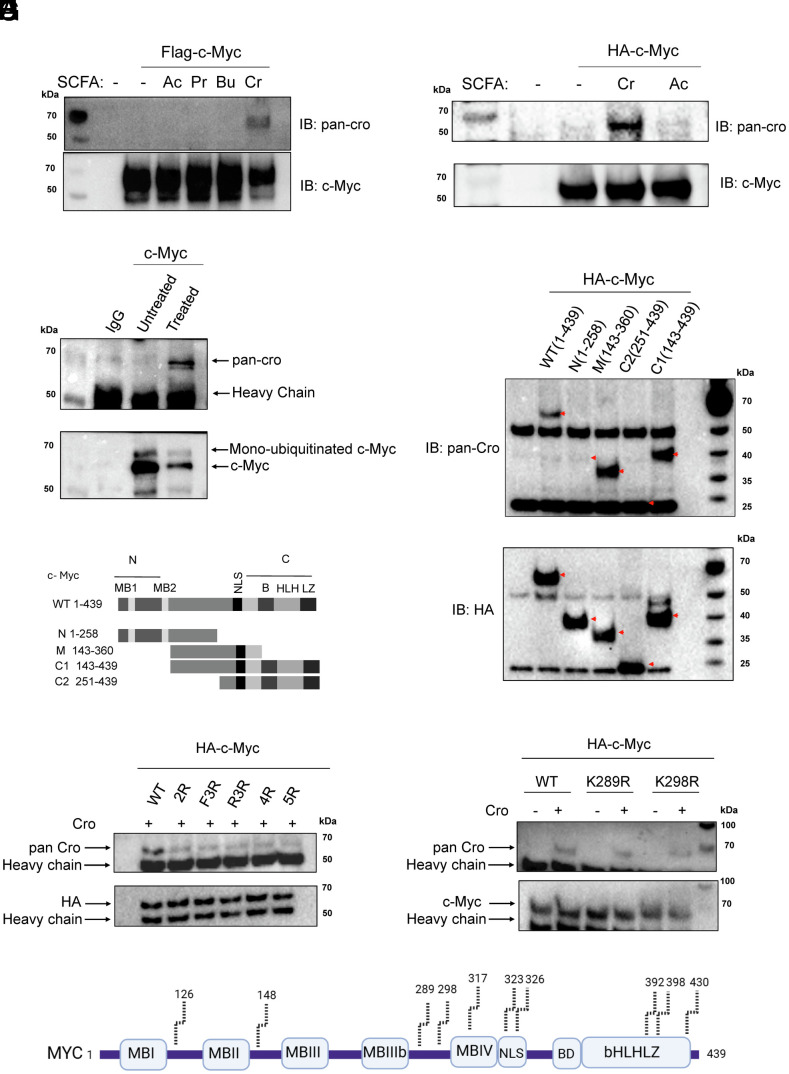
c-Myc is crotonylated in human cells. (*A* and *B*) Crotonylation of c-Myc in (*A*) HCT116 cells and (*B*) HEK293 cells was confirmed by WB analysis. Cells were transfected with Flag-c-Myc or HA-c-Myc for 48 h, treated with 10 mM short-chain fatty acids (acetate, propionate, butyrate, and crotonate) overnight, and immunoprecipitated c-Myc was probed on WB with pan-crotonylation antibody. (*C*) Crotonylation of endogenous c-Myc was confirmed by WB analysis. HCT116 cells were treated with 10 mM crotonate overnight, 10 µM MG132 for 5 h before harvesting, and c-Myc was immunoprecipitated and probed on WB with pan-crotonylation antibody. (*D*) Deletion mutants of c-Myc confirm crotonylation site(s) reside on middle to C terminus. HEK293 cells were transfected with HA-tagged c-Myc and four HA-tagged deletion mutants for 48 h, treated with 10 mM crotonate overnight, and HA-tagged protein was immunoprecipitated and probed on WB with pan-crotonylation antibody. (*E*) Map of full-length c-Myc protein domains (*Top*) as well as four deletion mutants (*Bottom*). (*E* and *F*) K289 and K298 are identified as critical c-Myc crotonylation sites on WB. 2R mutant (K289R and K298R) shows near complete diminishment of staining from pan-crotonylation antibody signal; additional mutations in F3R (K275R, K289R, K298R), R3R (K289R, K298R, K317R), 4R (K289R, K298R, K317R, K323R), 5R (K298R, K298R, K317R, K323R, K326R) mutants did not produce a further diminishment of antibody signal (*E*). Single point mutants K289R and K298R show partial diminishment of antibody signal (*F*). HEK293 cells were transfected with HA-tagged c-Myc proteins for 48 h, treated with 10 mM crotonate overnight, and immunoprecipitated HA-tagged protein was probed on WB with pan-crotonylation antibody (*G*). 10 crotonylation sites on c-Myc identified by high-resolution mass spectrometry. Crotonylation sites of c-Myc identified by HRMS are denoted on c-Myc protein map (*H*).

To test if c-Myc is crotonylated in cells, we performed a set of biochemical and cell-based assays. HCT116 cells were incubated overnight with the short-chain fatty acids, including acetate, propionate, butyrate, and crotonate. c-Myc protein was immunoprecipitated following overexpression and short-chain fatty acid exposure, and then probed in WB analysis with a pan-crotonylation antibody. Notably, c-Myc was crotonylated, only after exposure to crotonate, but not the other short-chain fatty acids (Fig. 1*A*). This result was reproduced in HEK293 cells (Fig. 1*B*), indicating that c-Myc crotonylation is not cell-specific, as further validated below. To ensure endogenous c-Myc is crotonylated, HCT116 cells were incubated overnight with crotonate, treated with MG132 before collection, and c-Myc was immunoprecipitated and probed on WB analysis with a pan-crotonylation antibody (Fig. 1*C*). As before, a positive pan-cro WB signal was observed for the crotonate treated cells. Interestingly, the band for c-Myc crotonylation was observed closer to the monoubiquitinated c-Myc band than the endogenous c-Myc band, indicating a shift in electrophoretic mobility in the presence of MG132, a proteasome inhibitor, potentially resulting from change in protein charge and/or conformation. To identify crotonylation target residues, we generated a set of short c-Myc fragments, including the N terminus 1–258 (N), middle 143–360 (M), C terminus and middle 143–439 (C1), and C terminus 251–439 (C2) (Fig. 1*E*). These mutants were expressed in HEK293 cells alongside the full length and exposed to crotonate overnight. WB analysis of the immunoprecipitated deletion mutants showed that c-Myc is crotonylated along the middle to C termini (aa 251–439) as evidenced by positive pan-cro staining on these mutants, but not the N-terminal fragment (aa 1–258) (Fig. 1*D*). We also generated and evaluated various mutants of c-Myc with multiple lysine to arginine point mutations. As a result, c-Myc proteins with both the K289R and K298R mutations showed near complete diminishment of the pan-cro WB signals regardless of additional lysine to arginine mutations (Fig. 1*F*). Assessment of individual K289R or K298R mutant c-Myc showed intermediate ablation of pan-cro signal (Fig. 1*G*). To validate the findings with the pan-crotonylation antibody, we performed HRMS analysis of immunoprecipitated c-Myc protein following exposure of HEK293 cells to crotonate and pan-HDAC inhibitors (trichostatin A and nicotinamide). The complete c-Myc sequencing results are available in *SI Appendix*, Table S1 and the gel bands excised for sequencing are shown in *SI Appendix*, Fig. S1*A*. Consistently, the critical K289 and K298 residues as identified by WB analysis were detected by HRMS as crotonylation sites as well. Also, eight additional lysine residues were found to be crotonylated (Fig. 1*H* and *SI Appendix*, Table S1). Because the N terminus (aa1-258) of c-Myc was not crotonylated as detected by WB analysis with pan anti-crotonylation antibodies (Fig. 1*D*), K126 is less likely a crotonylation target residue. Thus, an 8R c-Myc mutant was generated encompassing the 2 critical (K289 and K298) and six additional lysines (K148, K317, K326, K392, K398, K430) that are substituted with 8 arginines (8R mutant). This 8R c-Myc mutant was evaluated using an in vitro chemical assay previously utilized by our laboratory. Mutation of the eight lysine residues to arginine resulted in significant loss of HRP-streptavidin signal compared to the wild type (*SI Appendix*, Fig. S1*B*). Taken together, these results demonstrate that c-Myc is indeed crotonylated in human cells.

### Crotonylation-Deficient c-Myc Mutants Exhibit Increased Proliferation-Promoting Capacity.

**Fig. 2.**
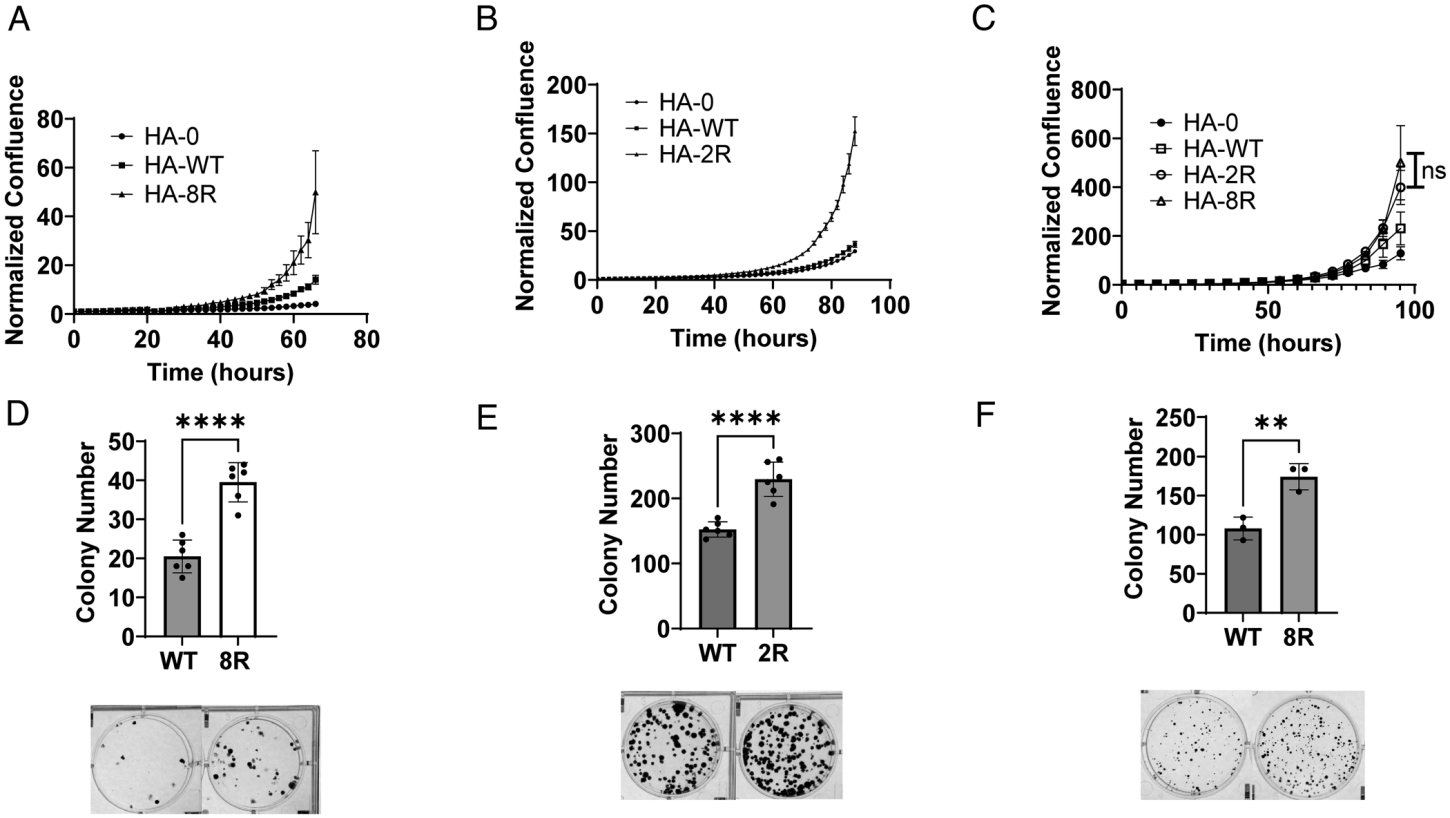
Crotonylation-deficient c-Myc mutants exhibit increased ability to promote short-term and long-term cell growth. (*A*–*C*) Short-term proliferation assays with H1299 (*A*), HEK293 (*B*), and HCT116 (*C*) stably transfected with HA-tagged empty vector or c-Myc protein demonstrate crotonylation-deficient mutant induces increased proliferative capacity of cells over short term (3 to 4 d). (*D*–*F*) Colony formation assay of H1299 (*D*), HEK293 (*E*), and HCT116 (*F*) stably transfected with HA-tagged empty vector or c-Myc protein demonstrate crotonylation-deficient mutants induce increased proliferative capacity over long term (7 to 10 d).

To determine if crotonylation might regulate c-Myc function, we performed a set of cell-based assays with crotonylation-deficient mutant c-Myc. First, the 8R c-Myc mutant was stably expressed in p53-deficient H1299 cells and evaluated in a short-term proliferation assay against control cells expressing the wild type c-Myc or empty vector (Fig. 2*A*). Interestingly, the 8R mutant cells showed significantly increased proliferation compared to control cells. Next, the 2R c-Myc mutant was evaluated similarly in HEK293 and H1299 cells, and the double mutant also showed a significant promoting effect on short-term proliferation of both the cells (Fig. 2*B* and *SI Appendix*, Fig. S2*A*). Additionally, the effects of 8R and 2R mutants on proliferation were directly compared in p53-deficient HCT116 cells; surprisingly, they showed very similar activity (Fig. 2*C*). This result was also reproduced in p53 mutant-harboring MDA-MB-231 cells (*SI Appendix*, Fig. S2*B*). Long-term proliferation was assessed in colony formation assays, and both the 8R and 2R mutants conferred increased colony formation in H1299, HEK293, and p53-null HCT116 cells (Fig. 2 *D*–*F*). These data confirm that mutation of the crotonylated lysine residues confers an increase in cell proliferation, both short (cell proliferation) and long term (colony formation), and also highlights the significance of the two key crotonylation residues K289 and K298 in producing that effect. This increased effect was p53-independent, as it was observed regardless of the p53 status.

### Crotonylation-Deficient c-Myc Mutants Exhibit Increased Transcriptional Activity.

**Fig. 3.**
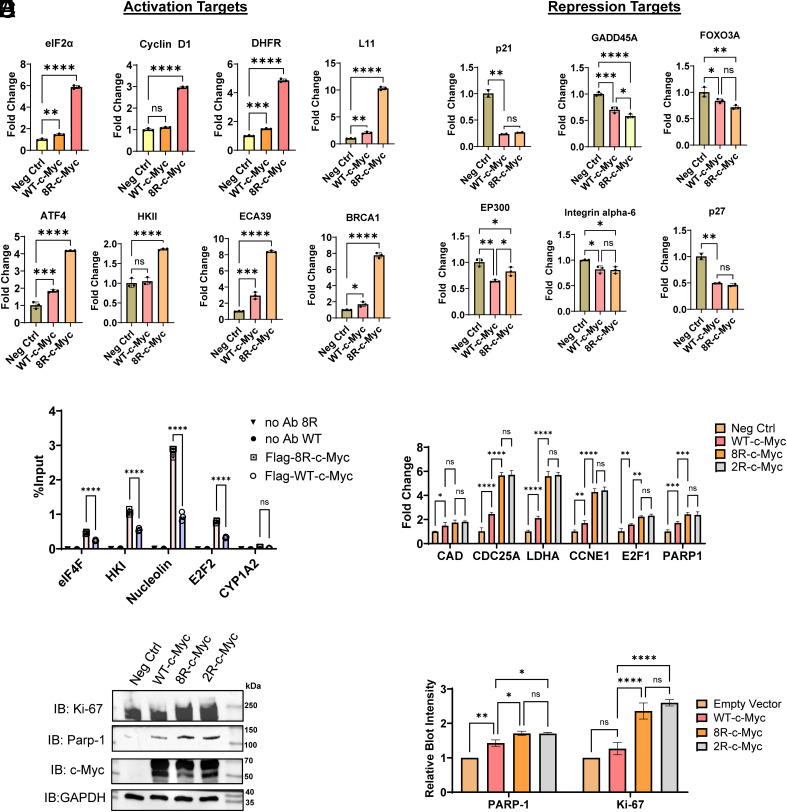
Crotonylation-deficient c-Myc mutants show increased transcriptional activity. (*A*) 8R mutant demonstrates increased c-Myc target gene activation by qPCR relative to wild type c-Myc and empty vector. All experiments were performed in stably transfected H1299 cells. (*B*) Repression targets of c-Myc were compared in H1299 cells transfected with empty vector, wild type, and 8R mutant but either no or minimal differences compared to activation targets were observed. (*C*) CHIP-qPCR was performed on H1299 cells transfected with Flag-tagged wild type c-Myc and 8R mutant. 8R mutant demonstrated increased promoter occupancy for tested genes. No antibody controls showed minimal amplification and CYP1A2 was used as negative control gene for c-Myc, which also showed nonsignificant amplification. (*D*) Target gene activation between 2R and 8R crotonylation-deficient c-Myc mutants showed no statistical difference on qPCR between the two but a significant increase compared to H1299 wild type c-Myc and empty vector stable cell lines. (*E*) H1299 stable cell lines used in (*A* and *D*) were used to show similar target gene activation of Parp-1 on WB by two cro-deficient c-Myc mutants as well as increased Ki-67 staining compared to wild type and empty vector cells. (*F*) Parp-1 and Ki-67 protein levels from (*E*) were measured using ImageJ after three replicate experiments were conducted.

To determine if the increased ability of c-Myc mutants to enhance cancer cell proliferation is attributed to their increased transcriptional activity, we first tested the expression of c-Myc downstream target genes involved in cell proliferation and survival (cell cycle, metabolism, ribosomal proteins, translation, integrated stress response, DNA repair, nucleotide and amino acid synthesis) by qPCR in 8R mutant stably expressed H1299 cells. Consistent with the results in Fig. 2, the 8R c-Myc mutant demonstrated significant induction of these downstream targets compared to wild type and empty vector expressing cells (Fig. 3*A*). Repression targets were also compared, but no statistically significant differences between the wild type and 8R were found, compared to that observed in the activation targets (Fig. 3*B*). Since binding to its obligate heterodimer partner Max is essential for c-Myc transcriptional activity (36), this was assessed by IP as some of the mutations (K392R, K398R, and K430R) reside in the Max-binding bHLHLZ domain of c-Myc. As a result, no apparent difference in Max binding was observed (*SI Appendix*, Fig. S3*A*). Subcellular localization was also assessed by confocal microscopy as one of the mutations K326R is in the c-Myc nuclear localization signal (NLS) domain. As observed, both the wild type and 8R mutant c-Myc were localized in the nucleoplasm of H1299 cells (*SI Appendix*, Fig. S3*B*). Next, we tested the ability of the mutant c-Myc to bind to the promoters of several c-Myc target genes critical for cell division, metabolism, ribosomal biogenesis, and translation by performing a CHIP-qPCR assay (Fig. 3*C*). Indeed, we found increased promoter occupancy by the 8R mutant compared to the wild type. To ascertain whether the 2R mutant transactivated c-Myc targets similarly to the 8R mutant, qPCR experiments examining genes in similar categories as before were performed. As a result, the 2R and 8R mutants displayed a similar activity to induce the expression of c-Myc target genes without significant differences between them (Fig. 3*D*). Finally, we compared both the 2R and 8R c-Mycs with their wild type counterpart directly by examining the expression of Ki-67, a proliferation marker, and Parp-1, a c-Myc target tested in Fig. 3*D*, via a WB analysis (Fig. 3*E*). Again, two c-Myc mutants more significantly enhanced the expression of the two genes at protein levels than did wild type c-Myc in H1299 cells that stably expressed each of the c-Myc variants (Fig. 3 *E* and *F*). Taken together, these results demonstrate that mutations of crotonylation target lysines, particularly K289R and K298R, empower the c-Myc mutants with significantly elevated transcriptional activity, particularly for those c-Myc activated target genes, without altering c-Myc’s nuclear import and ability to bind to its partner Max.

### Crotonylation-Deficient c-Myc Mutants Bind Strongly to SKP2.

**Fig. 4.**
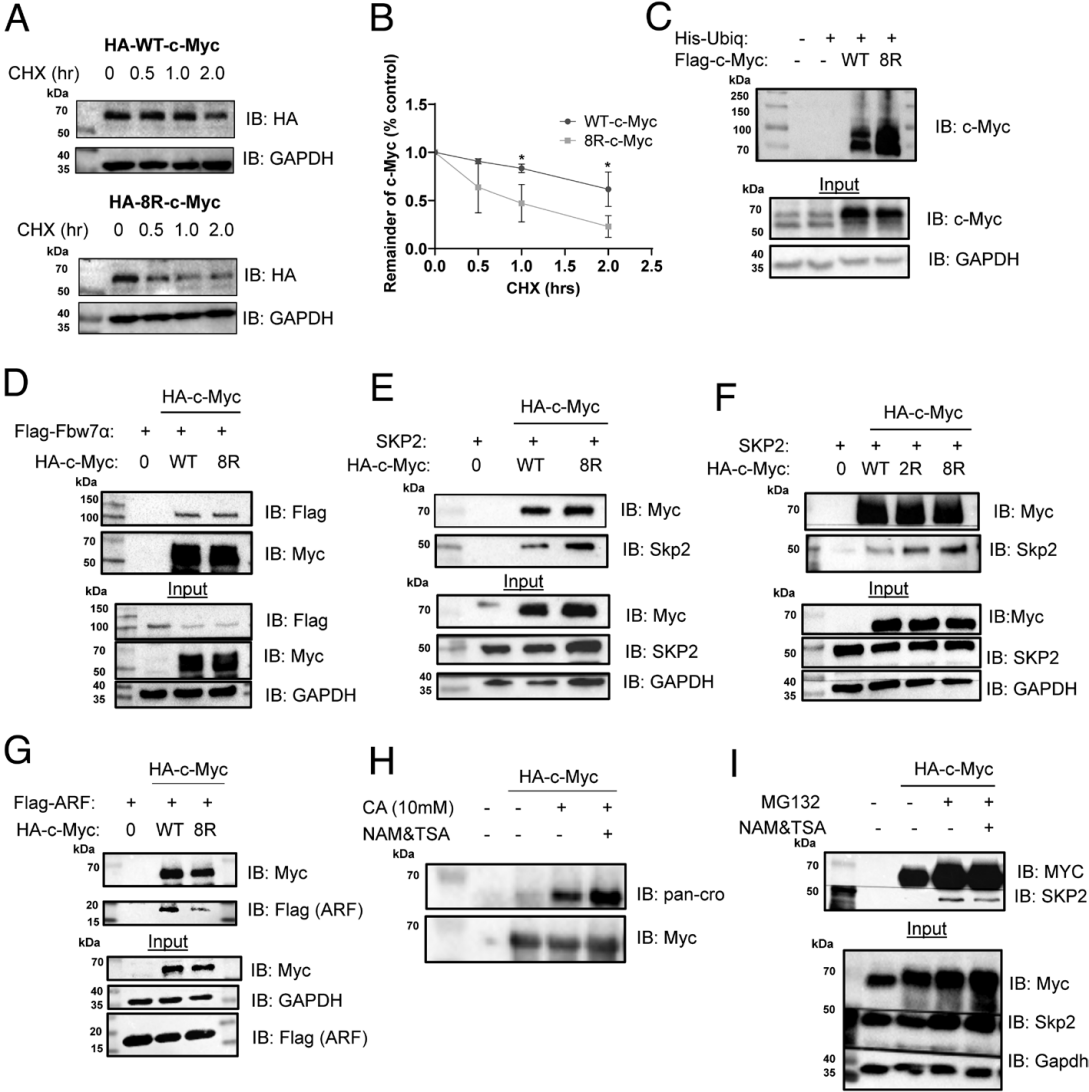
Crotonylation-deficient c-Myc mutants exhibit shorter half-life and increased SKP2 binding. (*A*) 8R mutant showed a significant decrease in half-life relative to wild type. Cycloheximide chase was performed on H1299 cells transfected with HA-tagged wild type c-Myc and 8R c- mutant after 48 h using time points of 0, 0.5, 1.0, and 2.0 h. Exogenous protein decay was detected by WB using HA antibody. (*B*) HA-c-Myc protein levels from (*A*) were measured using ImageJ after three independent experiments were conducted. (*C*) Ubiquitination assay showed 8R mutant is more ubiquitinated compared to wild type c-Myc. H1299 cells were transfected with Flag-tagged c-Myc proteins and His-ubiquitin for 48 h, MG132 was added at 20 μM for 6 h, cells were harvested for immunoprecipitation with nickel beads, and WB was performed with appropriate controls. (*D*) Co-IP and WB analysis showed that wild type c-Myc and 8R mutant exhibit similar binding to Fbw7α. HEK293 cells were transfected with Flag-tagged Fbw7α and HA-tagged c-Myc proteins for 48 h, treated with MG132 for 4 h prior to harvesting, followed by HA bead pull-down and WB analysis with the specified antibodies. (*E*) Co-IP and WB analysis showed that 8R mutant binds more strongly to SKP2 than wild type c-Myc. HEK293 cells were transfected with SKP2 and HA-tagged c-Myc proteins for 48 h, treated with MG132 for 4 h prior to harvesting, followed by HA bead pull-down and WB analysis with the specified antibodies. (*F*) Co-IP and WB analysis showed that 2R and 8R mutants exhibit increased binding to SKP2 compared to wild type c-Myc. H1299 cells were transfected with SKP2 and HA-tagged c-Myc proteins for 48 h, treated with MG132 for 4 h prior to harvesting, followed by HA bead pull-down and WB analysis with the specified antibodies. (*G*) Co-IP and WB analysis showed that wild type c-Myc binds to p14ARF more strongly than 8R mutant. H1299 cells were transfected with Flag tagged-p14ARF and HA-tagged c-Myc proteins for 48 h, followed by HA bead pull-down and WB analysis with the specified antibodies. (*H*) IP and WB analysis showed pan-HDAC inhibitors TSA (10 nM) and nicotinamide (5 mM) increased c-Myc crotonylation. HEK293 cells were transfected with HA-tagged c-Myc protein for 48 h, treated with crotonate and HDAC inhibitors overnight, and followed by HA bead pull-down and WB analysis with pan-crotonylation antibody. (*I*) Co-IP and WB analysis showed that SKP2 binding to c-Myc decreases upon the same HDAC inhibitor treatment from (*H*). HEK293 cells were transfected with SKP2 and HA-tagged c-Myc proteins for 48 h, treated with crotonate and HDAC inhibitors overnight, treated with MG132 for 4 h prior to harvesting, followed by HA bead pull-down and WB analysis with the specified antibodies.

To elucidate the mechanism(s) of how the c-Myc 2R and 8R mutants gain stronger transcriptional activity than does their wild type counterpart, we compared their half-lives by transiently expressing each of the wild type and 8R mutant c-Mycs in H1299 cells. Surprisingly, the mutant c-Myc showed a shorter half-life than did the wild type c-Myc (Fig. 4 *A*-*B*). As c-Myc protein turnover is well known to be tightly regulated by the ubiquitin proteasome system (UPS), we conducted a ubiquitination assay and found a clear increase in ubiquitination for the mutant c-Myc, compared to the wild type c-Myc (Fig. 4*C*). Because Fbw7 was previously shown to ubiquitinate c-Myc by binding to its N terminus (*SI Appendix*, Fig. S4*A*) we first tested if mutant c-Myc might bind to this ubiquitin ligase better than does wild type c-Myc by performing IP-WB assays in H1299 and HEK293 cells (37, 38). However, no increase in binding by the 8R mutant was observed compared to the wild type c-Myc in either of the cells (Fig. 4*D* and *SI Appendix*, Fig. S4*B*). This was consistent with the fact that Fbw7 binds to the N terminus of c-Myc, where no crotonylation site was identified and thus no mutation was made (*SI Appendix*, Fig. S4*A*). Next, considering the increased activity of the mutant and the overlap between c-Myc’s Skp2-binding and crotonylation target lysines-harboring domains (*SI Appendix*, Fig. S4*A*), we tested their binding to Skp2 in HEK293 and H1299 cells that ectopically expressed Ha-c-Myc and Skp2 by IP-WB analysis. Interestingly, anti-Ha antibodies pulled down more Skp2 proteins with the 8R mutant than that with the wild type c-Myc (Fig. 4 *E* and *F*). Again, both the 2R and 8R c-Myc mutants showed a similar level of increase in binding to Skp2 (Fig. 4*F*). This suggested a mechanism by which the increased activity of these mutants might be attributed to their increased binding to Skp2, a ubiquitin ligase responsible for both the activation and the postactivation proteolysis of c-Myc (18, 19).

To test this hypothesis, we conducted an IP-WB analysis for the 8R and wild type c-Mycs with p14ARF, a negative regulator of c-Myc activity previously shown to bind c-Myc competitively with Skp2 (39). As expected, the mutant bound much less to ARF than did the wild type c-Myc (Fig. 4*G*). This finding was consistent with the minimal effect of the mutant c-Myc on the expression of c-Myc’s repression target genes (Fig. 3*B*), as ARF was previously shown to primarily affect c-Myc’s activation activity (37, 38). Finally, we tested if crotonylation of c-Myc might affect formation of the Skp2–c-Myc complex by conducting an IP-WB assay with pan HDAC inhibitors (trichostatin A and nicotinamide) added to HEK293 cells, a mixture which increases the pan-crotonylation signal of c-Myc as detected by WB analysis with anti-Pan crotonylation antibody (Fig. 4*H*). As shown in Fig. 4*I*, the treatment of the cells with this mixture reduced the formation of the endogenous wild type c-Myc–Skp2 complex. To test if crotonic acid might affect the proliferation, growth, and c-Myc target expression in cancer cells, we conducted CCK8, colony formation, and qPCR assays after overnight treatment with sodium crotonate in H1299 cells. As expected, this treatment significantly reduced the short term cell survival (*SI Appendix*, Fig. S4*A*) and long term colony formation (*SI Appendix*, Fig. S4*B*) of the cancer cells as well as the expression of several c-Myc target genes (*SI Appendix*, Fig. S4*C*). Together, these results indicate that crotonylation of c-Myc suppresses its transcriptional activity, consequently inhibiting its function to promote cancer cell growth and survival, while mutations of its two key crotonylation residues, K289R and K298R, rescue the impaired cellular functions inversely.

### Cancer-Derived c-Myc K298N Mutant Possesses Increased Transcriptional Activity and Oncogenic Potential.

**Fig. 5.**
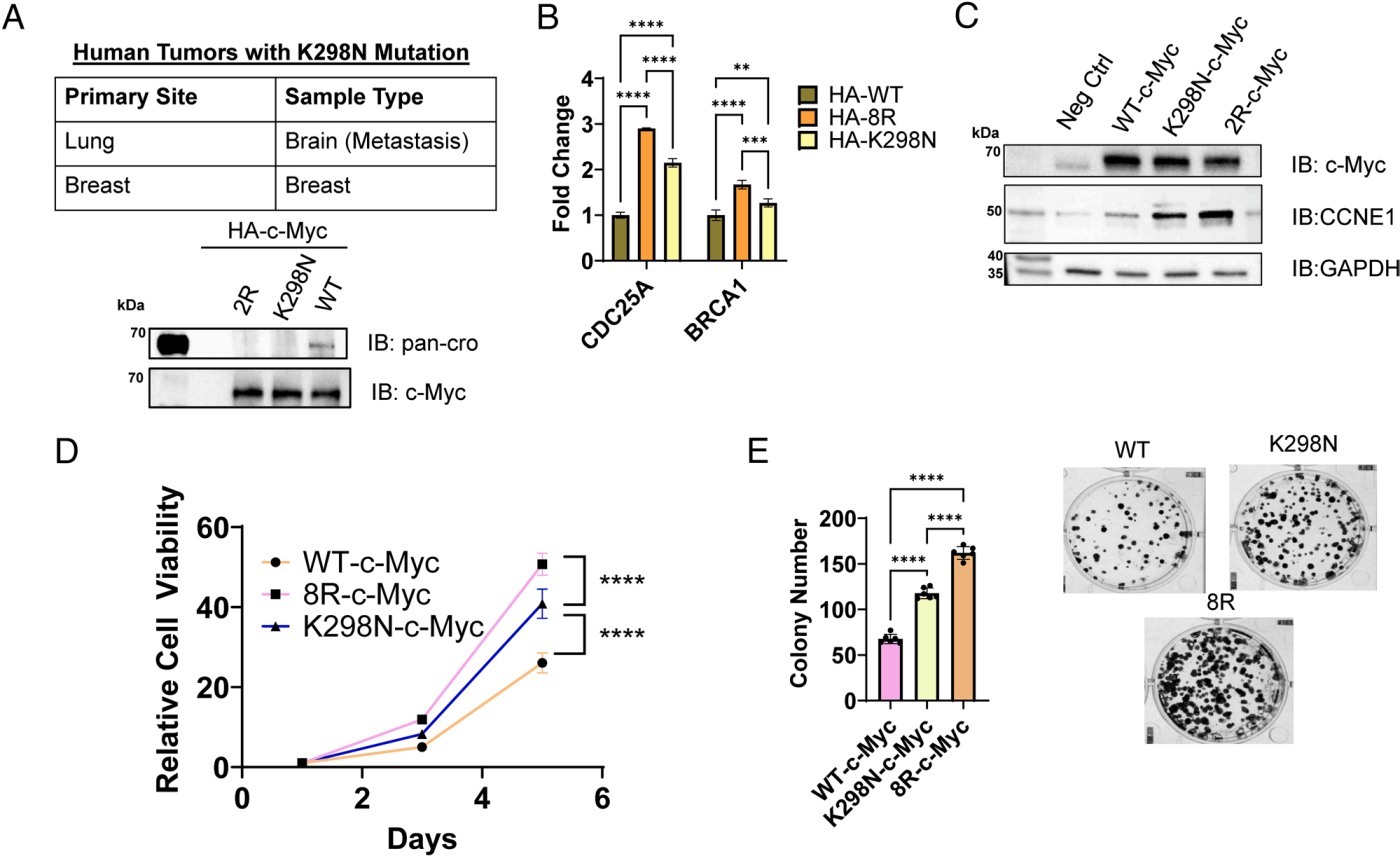
Cancer-derived K298N c-Myc mutant demonstrates increased transcriptional activity and oncogenic potential. (*A*) Two independent cases of c-Myc K298N mutation were identified in databases of tumor genetic sequences. WB analysis of immunoprecipitated c-Myc in HCT116 shows a loss of pan-crotonylation staining from K298N mutant relative to wild type, similar to 2R. (*B*) qPCR of H1299 cell pools transfected with WT c-Myc, 8R-cMyc, and K298N-cMyc shows increased activation of downstream targets in mutants relative to wild type. (*C*) WB analysis of H1299 cell pools transfected with WT c-Myc, K298N c-Myc, and 2R c-Myc shows an increased activation of downstream target CCNE. (*D*) CCK8 analysis of H1299 cell pools from (*B*) showed an increase in short-term viability for both mutants relative to WT. (*E*) Colony formation assay of H1299 cell pools from (*B*) showed an increase in long-term viability for both mutants relative to WT.

To further show the clinical relevance of these crotonylated lysine residues in affecting c-Myc oncogenic functions, we analyzed a human tumor database for the presence of possible mutations at either of the two significant crotonylation residues. Interestingly, two patients with K298N point mutation were identified (Fig. 5 *A*, upper table). This mutant was also shown with the pan-crotonylation antibody to have reduced crotonylation staining on WB compared to wild type, similar to 2R mutant, when overexpressed in p53-deficient HCT116 cells (Fig. 5 *A*, *Lower*). The transcriptional activity of the K298N mutant was compared to the wild type and 8R mutant in qPCR analysis. Consistent with the result in Fig. 3, this mutant significantly induced the expression of c-Myc target genes compared to wild type c-Myc, though to a less degree compared to the c-Myc 8R mutant (Fig. 5*B* and *SI Appendix*, Fig. S5*A*). This was also confirmed by WB analysis using H1299 cell pools expressing wild type and mutant c-Myc proteins as the K298N mutant more drastically elevated CCNE1 expression compared to wild type c-Myc, though to a less degree compared to the c-Myc 2R mutant (Fig. 5*C*). Again, the K298N mutant more significantly promoted cell proliferation and colony formation than did wild type c-Myc, though to a less degree compared to the 8R mutant, when overexpressed in H1299 cells (Fig. 5 *D* and *E*). These results demonstrate that the cancer-derived c-Myc K298N mutant possesses a stronger transcriptional and oncogenic function compared to its wild type counterpart.

### Cancer-Derived c-Myc K298N Mutant Gains More Oncogenic Function In Vivo.

**Fig. 6.**
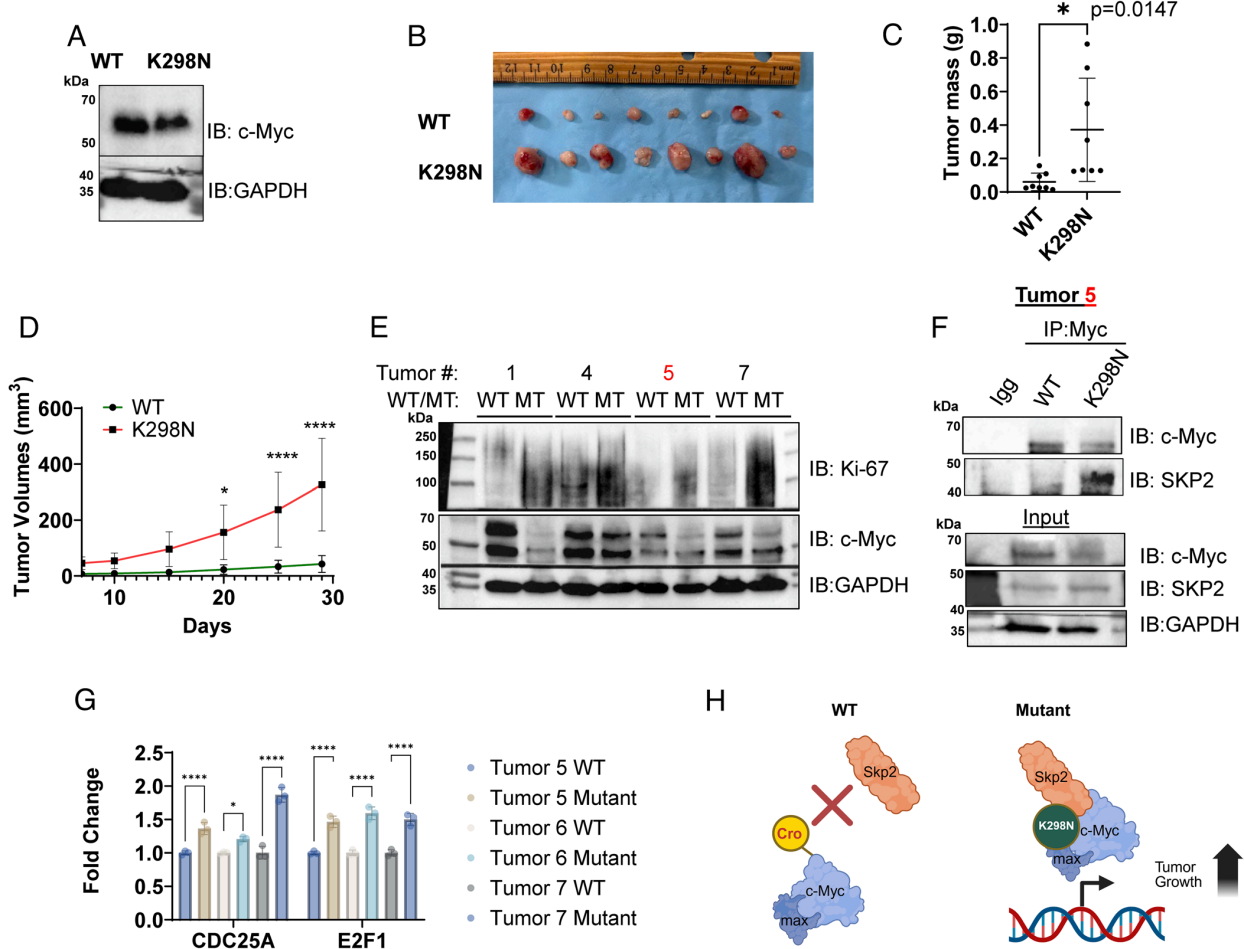
Cancer-derived K298N c-Myc mutant possesses more oncogenic function in vivo. (*A*) WB analysis of H1299 cells stably transfected with WT and K298N c-Myc show similar expression levels prior to subcutaneous injection into eight nude mice for xenograft experiment. (*B*–*D*) Xenograft tumors were harvested after 4 wk and K298N tumors exhibited increased tumor growth as evidenced in image of paired tumors (*B*), tumor weights (*C*), and tumor volumes measured every 3 to 5 d with digital calipers (*D*). (*E*) WB analysis of ground tumor tissue from four mice shows expression levels of c-Myc in tumor pairs similar to as before injection (*A*) and an increase in Ki-67 stain for mutant relative to WT. A section of tissue sample was removed from tumor and lysed in RIPA buffer with a Dounce homogenizer and prepared for WB analysis. (*F*) Co-IP and WB analysis showed that K298N mutant exhibit increased binding to SKP2 compared to wild type c-Myc in ground tissue from tumor 5. A section of tissue sample was removed from tumor and lysed in RIPA buffer with a Dounce homogenizer and prepared for Co-IP and WB analysis. (*G*) qPCR of c-Myc downstream targets from three tumor pairs shows an increase in activation for K298N mutant relative to WT. A section of tissue sample was removed from each tumor and lysed in Trizol buffer with a Dounce homogenizer and prepared for qPCR analysis. (*H*) Working model generated in bioRender depicts crotonylation at K298 inhibiting binding of SKP2 to c-Myc preventing tumor growth, whereas in the mutant this inhibition is relieved and tumor growth promoted. The Student’s paired *t* test was used to present mean differences among groups. Data are represented as mean ± SD. *P < 0.05, **P < 0.01, ***P < 0.001, ****P < 0.0001 vs. control.

To determine the oncogenic potential of the cancer-derived K298N mutant in vivo, we generated a mouse xenograft tumor model system by injecting p53-deficient H1299 cells that stably expressed either wild type or K298N c-Myc into nude mice. Their expression was verified by WB analysis before rear flank injection of the cells into nude mice (Fig. 6*A*). Tumors were harvested after 4 wk and K298N tumors demonstrated an increase in size when compared side by side to wild type tumors (Fig. 6*B*). Remarkably, tumor mass and volume of the xenograft human lung tumors that harbor the K298N mutant were significantly larger and greater than were those of the wild type c-Myc-harboring tumors, as compared using paired *t* test statistical analysis (Fig. 6 *C* and *D*). WB analysis showed a marked increase of Ki-67 protein levels in the mutant c-Myc-containing tumors compared to those with wild type c-Myc (Fig. 6*E*). Of note, the level of the mutant c-Myc protein was relatively lower than of wild type c-Myc (Fig. 6*E*); this was likely due to the less stable nature of the mutant c-Myc than that of the wild type c-Myc (*SI Appendix*, Fig. S5*B*) and the increased binding of the mutant to Skp2 compared to the wild type in the tumors (Fig. 6*F*). Once again, this K298N mutant significantly increased the expression of some c-Myc target genes as tested by qPCR analysis (Fig. 6*G*). Taken together with the results in Fig. 5, these results demonstrate that the cancer-derived native c-Myc mutant K298N gains much stronger oncogenic functions by interacting with Skp2 and boosting its transcriptional activity, consequently promoting tumorigenesis (Fig. 6*H*).

## Discussion

Although c-Myc has been intensively studied with multiple PTMs, our study as presented here unveils a novel acylation of c-Myc, called crotonylation, which we show significantly influences the transcriptional activity of this key oncoprotein (9, 40). We identified a total of potential 10 crotonylated lysine residues by HRMS (Fig. 1*G* and *SI Appendix*, Fig. S1*A* and Table S1). Through a combination of mutagenesis, WB, qPCR, and cell-based assays, we narrowed down two lysine residues as key players for conferring a unique mutant phenotype to be K289 and K298 (Figs. 1–4 and *SI Appendix*, Figs. S1–S4). Interestingly, our studies reveal that crotonylation at these specific residues significantly impacts c-Myc ubiquitination and turnover as well as transcriptional activity, consequently inducing cell proliferation and survival, two of this protein’s key functions in promoting oncogenic progression, through a unique mechanism involving recruitment of the E3 ligase Skp2 (Fig. 4). Remarkably, one of the two key residues, K298, was mutated to Asparagine (N) in two cases of primary human lung and breast cancers. Through both cell-based assays and a xenograft mouse model system, we further demonstrated that this K298N mutation also confers an increase in c-Myc activity and subsequent tumorigenesis using a similar mechanism as do the c-Myc 2R and 8R mutants (Figs. 5 and 6). This work represents a significant contribution to the current knowledge of c-Myc regulation via PTMs in literature for the following reasons.

First, these two lysine residues, which, once mutated, confer cells with a significant proliferative advantage, have not been previously identified as targets for c-Myc acylation, and thus our finding is extremely novel from this perspective. Second, the mechanism proposed herein by which the crotonylation of these two lysine residues hinders the binding of ubiquitin ligase Skp2, which is necessary for not only the proteolysis of c-Myc but also the activation of many of its downstream targets, is also a step forward in the field because it represents a yet untested avenue of exploration by which acylation of c-Myc could affect its turnover and activity (Figs. 1–4 and 6*H*). Last, the identification of tumors bearing K298N mutation at one of the significant lysine crotonylation residues as well as our xenograft mouse model validates the importance of this lysine in effecting changes in c-Myc transcriptional activity (Figs. 5 and 6).

c-Myc activity is aberrantly dysregulated in the majority of cancer types (41). The upregulation in c-Myc transcriptional activity occurs at multiple nodes in tumors. Crotonylation presents a new regulatory pathway, by which the activity of c-Myc is modulated in tumors, and also a new avenue to explore for its pharmacological targeting. Crotonylation was initially discovered as an evolutionarily conserved histone modification, but its role has since quickly expanded as a significant modification to a host of nonhistone proteins with roles in metabolism, stem cell differentiation, DNA repair, and cell cycle progression (30, 31, 42). While the upstream pathways and enzymes governing crotonylation remain largely unexplored, what is well established is that the intracellular concentration of crotonyl-CoA is the primary driver of crotonylation, as addition of crotonate, converted to crotonyl Co-A by acyl-CoA synthetase short-chain family member 2 (ACSS2), pronouncedly raises crotonylation levels in the cell, as shown in the literature and this work (32, 43, 44). Significantly, cells can acquire crotonate from their extracellular environment; crotonic acid, a short-chain fatty acid, is generated by the gut microbiota through fermentation and, through passive diffusion as well as certain membrane transporters, can enter either the cells of the colon or other cells in the body after circulation (45). While the full range of crotonate-producing bacteria is still being researched, some species have been explicitly noted in the literature, such as *Acidaminococcus fermentans*, *Emergencia timonensis*, and a variety of Clostridia bacteria which are well known for their butyrate synthesis pathways that generate crotonate as an intermediate (45–47). Moving forward, it will be valuable to examine these interactions in a mouse model colonized with these specific bacterial strains. In addition, metabolic pathways involved in fatty acid-oxidation and lysine degradation pathways can produce crotonyl-CoA as an endogenous by-product in the mitochondria; and recent studies showing that proteins involved in mitochondrial metabolic pathways or mitochondrial fusion/fission are targets of crotonylation, support this (48, 49). In the future it will be interesting and enticing to test whether manipulating these metabolic pathways can also change c-Myc crotonylation status.

Skp2 is overexpressed in numerous cancers, serving several roles in promoting cell cycle entry, including degradation of key checkpoint inhibitors, but with respect to c-Myc, it has been shown to be critical in promoting the G1-S transition, at which their binding reaches a zenith before proteasomal degradation of c-Myc commences (50). Silencing of Skp2 or c-Myc leads to G1 cell cycle arrest, and numerous small molecule or small peptide inhibitors have been developed for both proteins and tested in preclinical models for several advanced stage cancer types (21, 51, 52). The identification of key crotonylation residues which serve to modulate the binding of these two oncoproteins, an essential step for cell cycle entry, presents not only a new interface for development of novel inhibitors but also a new approach to answer fundamental c-Myc biology questions. c-Myc promotes transcription of its specific target genes but also global transcription through facilitating the positive transcription elongation factor b (P-TEFb) complex formation (53). The mutants which we uncovered in our work can be used to determine not only which target genes are specifically affected by c-Myc and Skp2 interaction but also whether the complex formation of Skp2 and c-Myc has a role in promoting global transcription. Additionally, this mutant provides a helpful model in unwinding the long-standing paradox of Skp2 both activating c-Myc’s transcriptional activity and promoting its proteasomal turnover (Fig. 6*H*) (54). The current model points to Skp2 licensing c-Myc to activate gene transcription by ubiquitylation and rapid degradation. While studies show c-Myc can bind to DNA without ubiquitylation, its rapid turnover is essential for the recruitment of critical coactivators, such as Transformation/Transcription Domain-Associated Protein (TRRAP), and essential components of the RNA polymerase II (RNAPII) transcriptional elongation machinery, such as P-TEFb (55). The crotonylation-deficient mutants generated in our study will be useful in parsing out further mechanistic details of this transcriptional licensing process.

With regard to the detailed structural mechanism, in what manner c-Myc crotonylation inhibits Skp2 binding remains unclear. Alpha Fold predictions of c-Myc with the 10 lysine residues being crotonylated points to a far more compact, less open and less disordered structure (which would also be far easier to pharmacologically target as has been shown for other conformational switches of c-Myc (56) compared to the unmodified form (*SI Appendix*, Fig. S6). The intrinsically disordered and flexible structure of c-Myc is a benefit in many cases for it to be able to bind its many coactivators, which often require c-Myc to bind at disparate domains on the same coactivator, such as is the case for Skp2. Detailed structural studies would be necessary to more fully understand the changes in c-Myc–Skp2 interaction caused by crotonylation. This type of structural study may also yield information about the basis of the competitive binding of c-Myc by Skp2 and p14ARF (Fig. 4*G*), which is clearly influenced by the crotonylation of K289 and K298.

It is also important to emphasize the limitations of the classical mutagenesis studies this work relies upon. The observed phenotypes of the crotonylation-deficient mutants may very well be attributable to multiple PTMs being blocked by the substitution of lysine for arginine. Also, when mutations are introduced to a protein, changes in the primary structure can occur that may affect function. This study attempts to mitigate these concerns by focusing on two lysines, K289 and K298, which are two residues that, while in the region of c-Myc, which is well known for PTMs such as acetylation and SUMOylation, there is minimal literature describing modification of these two, but a significant proliferative advantage conferred upon their mutations.

Some of the other crotonylation sites in the 8R mutant are noted acetylation sites (K148, K317, K323) and SUMOylation sites (K148, K326, K392, K398, and K430); so although it is clear that there is some competition between these other PTMs and crotonylation, we desired to separate the effects as much as possible in this work (57, 58). Future studies will be necessary to untangle the effects of these unique PTMs. Ubiquitination also can occur on several lysines on the N and C termini of c-Myc. However, the fact that the 8R mutant displays increased ubiquitination (Fig. 4*C*) suggests that these crotonylation residues are unlikely critical ubiquitination target residues in this biological context, as previous work has pointed to Skp2 ubiquitylation of c-Myc occurring mainly on its N-terminal TAD (18). Immediate future work will focus on identifying the upstream pathways or enzymes, such as crotonyl transferase or decrotonylase, which facilitate increased or decreased c-Myc crotonylation, and understanding whether the PTM occurs via an enzymatic or nonenzymatic route. This would allow additional approaches to manipulate c-Myc crotonylation status in cell culture or in vivo.

## Materials and Methods

### Cell Culture and Transient Transfection.

Cells were cultured in Dulbecco’s modified Eagle’s medium (DMEM) supplemented with 10% FBS, 50 U/mL penicillin, and 0.1 mg/mL streptomycin at 37 °C in a humidified 5% CO_2_ atmosphere. Transfection was performed using TurboFect (Thermo Scientific) following the manufacturer’s protocol. Cells were harvested 48 to 72 h posttransfection for analysis. Cells used for this study are listed in *SI Appendix*, Table S2.

### Western Blotting (WB) and Immunoprecipitation (IP).

Cells were lysed in standard lysis buffer (50 mM Tris/HCl pH 7.5, 1 mM EDTA, 150 mM NaCl, 0.5% NP-40, 0.2 mM PMSF, 1 mM DTT, 1 mM leupeptin, 10 mM pepstatin A), and lysates were clarified by centrifugation. For IP, 500 to 1,000 µg of lysate was precleared with Protein A/G beads (Santa Cruz Biotechnology) and incubated with specific antibodies or HA-/Flag-conjugated beads at 4 °C for 6 h or overnight. Bead–protein complexes were washed with lysis buffer, RIPA buffer (50 mM Tris-HCl pH 7.4, 150 mM NaCl, 1% Triton X-100, 0.1% SDS, 1% sodium deoxycholate), or SNNTE buffer (50 mM Tris-HCl pH 7.4, 5 mM EDTA, 5% sucrose, 1% NP-40, 0.5 M NaCl), and bound proteins were eluted by boiling in SDS loading buffer for 8 to 15% SDS-PAGE analysis. Antibodies are listed in *SI Appendix*, Table S2.

### Generating Stable Cell Lines and Cell Pools.

Cells are transfected with plasmid containing both gene of interest and neomycin resistance gene. Following 24 to 48 h allowing cells to recover and begin expressing resistance gene, standard growth media are replenished with media containing an optimized concentration of G418 for each cell line. Over 7 to 14 d, media are replenished every 3 d to ensure a consistent G418 concentration allowing nontransfected cells to die and the survival and expansion of resistant clones. The remaining population constitutes the stable cell pool, which was used for downstream experiments. Individual clones were also expanded to form a stable cell line to be used in downstream experiments. Stable cell lines and cell pools are listed in *SI Appendix*, Table S2.

### Cell Proliferation Assay.

Cell proliferation was assessed using the IncuCyte S3 Live-Cell Analysis System (Essen Bioscience) or the CCK-8 Kit. Cells were seeded at 500 to 5,000 cells per well in 96-well plates. IncuCyte captured images every 3 h over 4 d, and data were analyzed using the Basic Analyzer module. For CCK-8, absorbance at 450 nm was recorded daily for 3 d using a SpectraMax M5e Microplate Reader (Molecular Devices).

### Colony Formation Assay.

Transfected cells were seeded in 6-well plates at 1,000 cells per well. Colonies were grown for 2 wk with media replacement every 2 to 3 d, fixed with 10% formalin, and stained with crystal violet. Colony numbers were quantified using ImageJ software.

### Immunofluorescence (IF) Staining Assay.

Cells were fixed with 4% paraformaldehyde or 10% formalin for 10 min, permeabilized with 0.2% Triton X-100 for 10 min, and blocked with 1% BSA for 30 min. Primary antibodies were incubated overnight at 4 °C, followed by Alexa Fluor-conjugated secondary antibodies (Invitrogen-Thermo) at RT for 1 h. Nuclei were stained with DAPI, and images were acquired using a Nikon TiE-2 confocal microscope.

### Reverse Transcription and Quantitative Real-Time PCR (RT-qPCR).

Total RNA was extracted using Trizol (Invitrogen-Thermo). Reverse transcription was performed with 1 µg of RNA, poly-(T)20 primers, and M-MLV reverse transcriptase (Promega). Quantitative real-time PCR (RT-qPCR) was conducted using SYBR Green Mix (BioRad) with primers provided in *SI Appendix*, Table S3.

### Chromatin IP-qPCR.

Confluent 15 cm dishes of H1299 cells, 72 h following transfection, were fixed by adding 1% formaldehyde to cell culture medium for 10 min at room temperature on slow shaker. Reaction was stopped by adding glycine to 0.125 M final concentration for 5 min at room temperature. Cells were harvested in cold PBS with PMSF. The CHIP procedure, as described previously (59), was used to isolate DNA with Flag antibody. Quantitative real-time PCR (RT-qPCR) was conducted using SYBR Green Mix (BioRad) with primers provided in *SI Appendix*, Table S4.

### PCR Mutagenesis.

c-Myc mutants were generated by site-directed PCR mutagenesis with primers from *SI Appendix*, Table S5 using a standard protocol with high-fidelity polymerase, DPN1 digestion, transformation, clone selection, and sequencing, as previously described (60).

### Co-IP and LC–MS Sample Preparation.

HA-c-Myc was overexpressed in HEK293 cells. Six hours following transfection, media were changed to either complete growth media or complete growth media containing 10 mM crotonic acid, 10 nM TSA, and 5 mM Nicotinamide. Thirty-six hours after transfection, cells were harvested, lysed in standard lysis buffer, and incubated overnight with anti-HA resin. After rinsing, bound proteins were eluted by boiling in SDS loading buffer for 8% SDS-PAGE. Following complete separation of bands at 125 V, gel was incubated in Imperial™ Protein Stain (Thermo Scientific) for 30 min following destaining in deionized water. The gel is shown in *SI Appendix*, Fig. S1*A*. The top bands (N1 and E1), corresponding to full-length c-Myc, were excised and destained in 25 mM NH_4_HCO_3_ with 50% acetonitrile (ACN). Gel pieces were reduced with dithiothreitol (DTT) and subsequently alkylated with iodoacetamide (IAA). The reduced and alkylated gels were then digested overnight with trypsin at 37 °C. The reaction was quenched with 20% formic acid (FA), and peptides were dried in a SpeedVac at 60 °C for 1 to 2 h. The dried peptides were reconstituted in LC–MS buffer (2% ACN and 0.1% FA in LC–MS–grade water) and transferred to LC–MS vials for LC–MS/MS analysis.

### LC–MS/MS Analysis and Data Processing.

Peptide samples were analyzed using a 100-min chromatographic method. The gradient consisted of 5 to 30% acetonitrile in 0.1% formic acid (ACN/FA) over 80 min, followed by 30 to 95% ACN/FA over 10 min. Chromatography was performed in a trap-and-elute configuration on an Ultimate 3000 HPLC system at a flow rate of 0.35 μL/min. The trap column was a PepMap C18 (5 μm, 300 μm × 5 mm), and peptides were separated on an EASY-Spray PepMap column (75 μm × 250 mm). Eluted peptides were analyzed on a Q Exactive HF-X Orbitrap mass spectrometer (Thermo Fisher Scientific). Survey scans were acquired in the Orbitrap at a resolution of 60,000 over an m/z range of 300 to 1,650, with an AGC target of 3 × 10^6 and a maximum injection time of 25 ms. Data-dependent MS/MS scans were acquired on precursors with charge states from +2 to +7, using higher-energy collisional dissociation (HCD) with a normalized collision energy of 27%. One full MS event was followed by 15 MS/MS events. Raw data were searched using PEAKS Studio v10.5 (Bioinformatics Solutions Inc., Waterloo, Canada) against the UniProt Homo sapiens reference proteome (Proteome ID: UP000005640). Search parameters were as follows: precursor mass tolerance, 20 ppm; fragment mass tolerance, 0.02 Da; up to two missed tryptic cleavages allowed. Carbamidomethylation of cysteine (+57.021 Da) was set as a fixed modification, while oxidation of methionine (+15.995 Da) and crotonylation of lysine (+68.0262 Da) were specified as variable modifications. Peptide identifications were filtered to achieve a false discovery rate (FDR) of 1%. The mass spectrometry proteomics data have been deposited to the ProteomeXchange Consortium via the PRIDE [1] partner repository with the dataset identifier PXD078303 and 10.6019/PXD078303 (61).

### Xenograft Tumor Assay Protocol.

Cells were trypsinized, counted, and aliquoted into PBS plugs (4.0 × 10^6^ cells/150 μL injection stored in 1 mL syringes) in tissue culture hood. Separate syringes transported on ice to animal facility. Mice were anesthetized with isoflurane inhalant just prior to injection. H1299 cells expressing wild type c-Myc were injected subcutaneously into mouse’s right flank and mutant cells injected subcutaneously into mouse’s left flank. Tumor growth was monitored every 3 to 5 d using digital calipers to calculate volume using formula V = (Width^2^ × Length) × 0.52. After 4 wk, mice were killed humanely and dissected to harvest tumor tissue. Tumors were weighed, measured, photographed, and frozen in liquid nitrogen for downstream analyses. This animal experiment was approved by the Institutional Animal Care and Use Committee at Tulane University School of Medicine.

### Quantification and Statistical Analysis.

ImageJ software was used to quantify colony formation assays and western blots. All experiments were conducted in triplicate. Group differences were evaluated using two-tailed Student’s *t* tests, with *P* < 0.05 considered statistically significant. Data are presented as mean ± SD.

## Supplementary Material

Appendix 01 (PDF)

Dataset S01 (PDF)

## Data Availability

Proteome data are available via ProteomeXchange with accession number PXD078303 (61). All other data are included in the article and/or supporting information.
