# Supplementary material for: Crotonylation impedes c-Myc oncogenic activity
Source: Proc Natl Acad Sci U S A. 2026 Jun 1;123(23):e2530020123. doi: 10.1073/pnas.2530020123 (PMC13250510; doi:10.1073/pnas.2530020123)
Supplement: Supplementary file 1 — Appendix 01 (PDF) [file pnas.2530020123.sapp.pdf]

Supplementary Information

Crotonylation Impedes c-Myc Oncogenic Activity

Nicholas J. Wallbillich[1], Peng Liao[1,2], Rashmi Srivastava[1,3], Jia Fan[1], Shelya X. Zeng[1*], and Hua Lu[1*]

[1] Department of Biochemistry & Molecular Biology and Tulane Cancer Center, Tulane University School of Medicine, 1430 Tulane Avenue, New Orleans, LA 70112, USA.

[2] Current address: Department of Surgery University of Michigan Medical Center, 2960 Aubman Health Sciences Library, 1135 Catherine, Ann Arbor, MI 48109, USA.

[3]Current Address: Laboratory of Translational Cancer Research, Ochsner Clinic Foundation, 1516 Jefferson Highway, New Orleans, LA70201, USA.

* Correspondence: Hua Lu, Department of Biochemistry & Molecular Biology and Tulane Cancer Center, Tulane University School of Medicine, New Orleans, LA 70112, USA. Phone: 504-988-0394

Email: hlu2@tulane.edu

* Correspondence: Shelya X Zeng, Department of Biochemistry & Molecular Biology and Tulane Cancer Center, Tulane University School of Medicine, New Orleans, LA 70112, USA. Phone: 504-988-3919

Email: szeng@tulane.edu

Author Contributions: Conceptualization, N.J.W., P.L, S.X.Z., and H.L.; methodology, N.J.W., P.L., J.F., S.X.Z., and H.L.; Investigation, N.J.W., P.L., R.S., G.M., J.F., S.X.Z. and H.L.; writing—original draft, N.J.W. and H.L.; writing—review & editing, N.J.W., S.X.Z., and H.L.; funding acquisition, H.L.; resources, J.F. and S.X.Z.; supervision, S.X.Z. and H.Lu..

Competing Interest Statement: The authors declare no competing interests.

Classification: Biological sciences, biochemistry

Keywords: c-Myc, Post-translational modification, crotonylation, Skp2, and oncogenesis.

.

This PDF file includes:

Main Text 1,1934 words
Figures 1 to X 6
Tables 1 to X 5

Supplemental 1

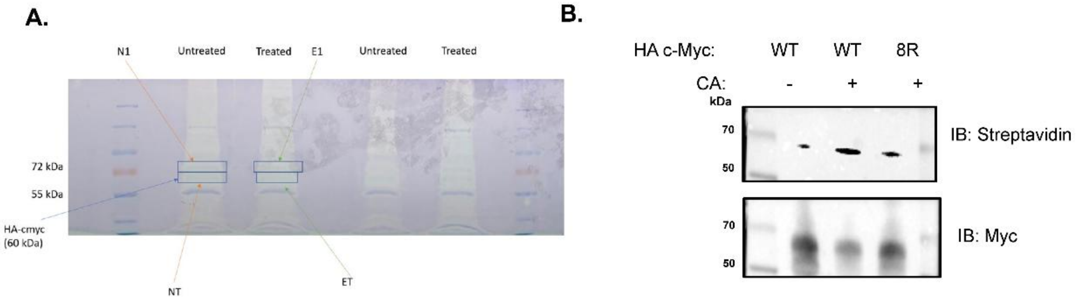

S. Figure 1. c-Myc is confirmed as a crotonylation target. (A) Image of Coomassie stained SDS-PAGE of immunoprecipitated c-Myc. N1 represents immunoprecipitated c-Myc from HEK293 cells with no treatment, and E1 represents immunoprecipitated c-Myc from HEK293 cells following treatment with 10 mM crotonate, 10 nM TSA, and 5 mM Nicotinamide. NT and ET are short-forms of c-Myc which were not analyzed by mass spectrometry **(B)** WB analysis following TCEP-Biotin chemical derivatization reaction with immunoprecipitated HA-tagged c-Myc protein from HEK293 cells given either no treatment or overnight crotonate treatment. Eight lysine to arginine mutations in 8R mutant was shown to diminish Streptavidin staining.

Supplemental 2

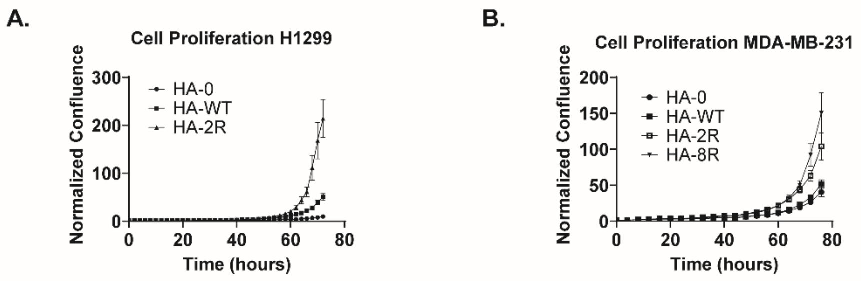

S. Figure 2. Crotonylation-deficient c-Myc mutants induce increased cell viability. **(A-B)** Cell proliferation assay with H1299 stable cell lines **(A)** and MDA-MB-231 cell pools **(B)** expressing empty vector, WT c-Myc, and 2R and/or 8R mutants show an increase in viability for cells expressing crotonylation-deficient mutants.

Supplemental 3

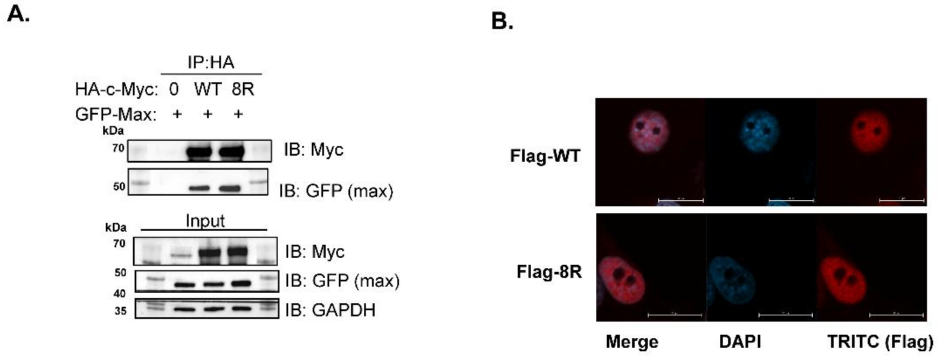

S. Figure 3. Crotonylation-deficient c-Myc mutants show increased downstream target activation. (A) Co-IP and WB analysis showed that wild type c-Myc and 8R mutant exhibit similar binding to max. H1299 cells were transfected with GFP-tagged max and HA-tagged c-Myc proteins for 48 h followed by HA bead pull-down and WB analysis with the specified antibodies. **(B)** Nuclear localization of c-Myc is maintained following mutation of crotonylated lysine residues. Flag-tagged WT and 8R c-Myc were transiently expressed in H1299 cells for 48 hours, formalin fixed, immunostained with the specified antibodies, and visualized using confocal microscopy. Scale bar: 20 μm.

Supplemental 4

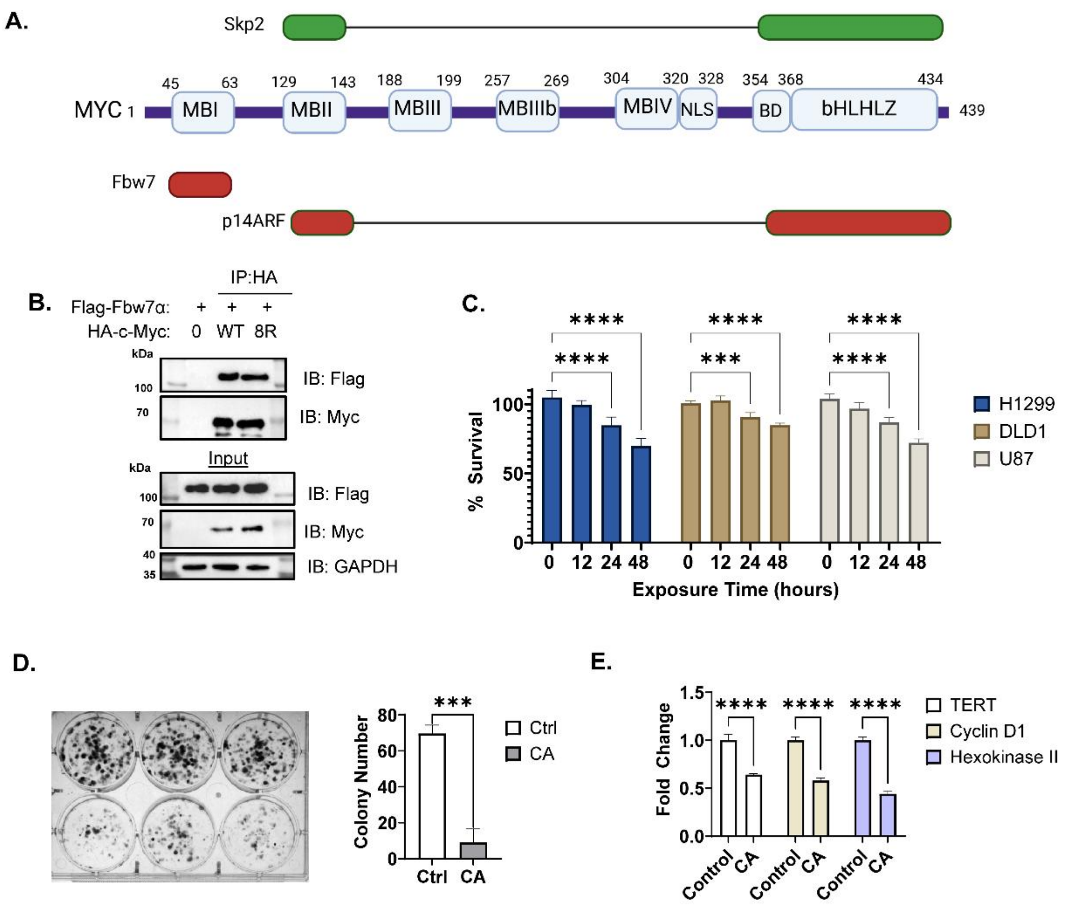

S. Figure 4 Crotonylation deficient mutants exhibit shorter half-life, increased SKP2 binding. (A) Binding domains of SKP2 and p14ARF show overlap with middle and c-terminus regions of c-Myc containing the crotonylated lysine residues. **(B)** Co-IP and WB analysis showed that wild type c-Myc and 8R mutant exhibit similar binding to Fbw7α. H1299 cells were transfected with Flag-tagged Fbw7α and HA-tagged c-Myc proteins for 48 h, treated with MG132 for 4 h prior to harvesting, followed by HA bead pull-down and WB analysis with the specified antibodies. **(C)** CCK8 time course analysis demonstrates that 10 mM crotonate treatment induces a decrease in short-term viability in the three indicated cell lines. **(D)** Colony formation assays demonstrate than treatment with 20 mM crotonate induces a decrease in long term viability of H1299 cells. **(E)** qPCR shows a decrease in c-Myc downstream target activation after overnight treatment of HCT116 cells with 10 mM crotonate, 10 nM TSA, and 5 mM nicotinamide.

Supplemental 5

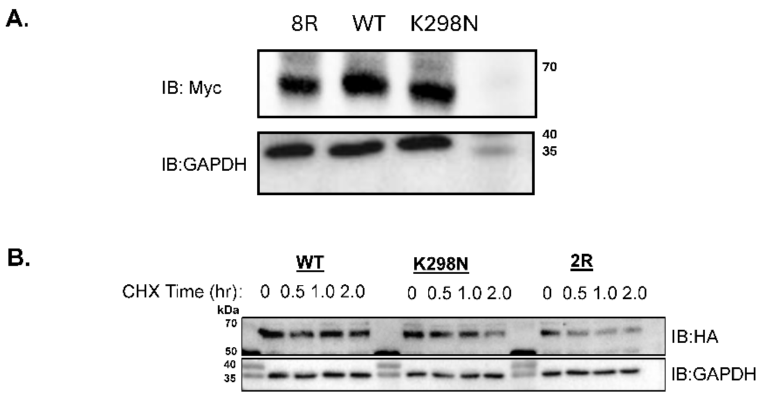

S. Figure 5. Cancer derived K298N c-Myc mutant also demonstrates increased activity. (A) WB analysis shows protein levels of wild type and mutant c-Myc in H1299 pools used to generate qPCR data in Figure 5B. **(B)** K298N and 2R mutants demonstrate decreased half-life relative to WT c-Myc. Cycloheximide chase was performed on H1299 cells transfected with HA-tagged wild type c-Myc, K298N c-Myc, and 2R c-Myc after 48 hours using time points of 0, 0.5, 1.0, and 2.0 hours. Exogenous protein decay was detected by WB using HA antibody.

Supplemental 6

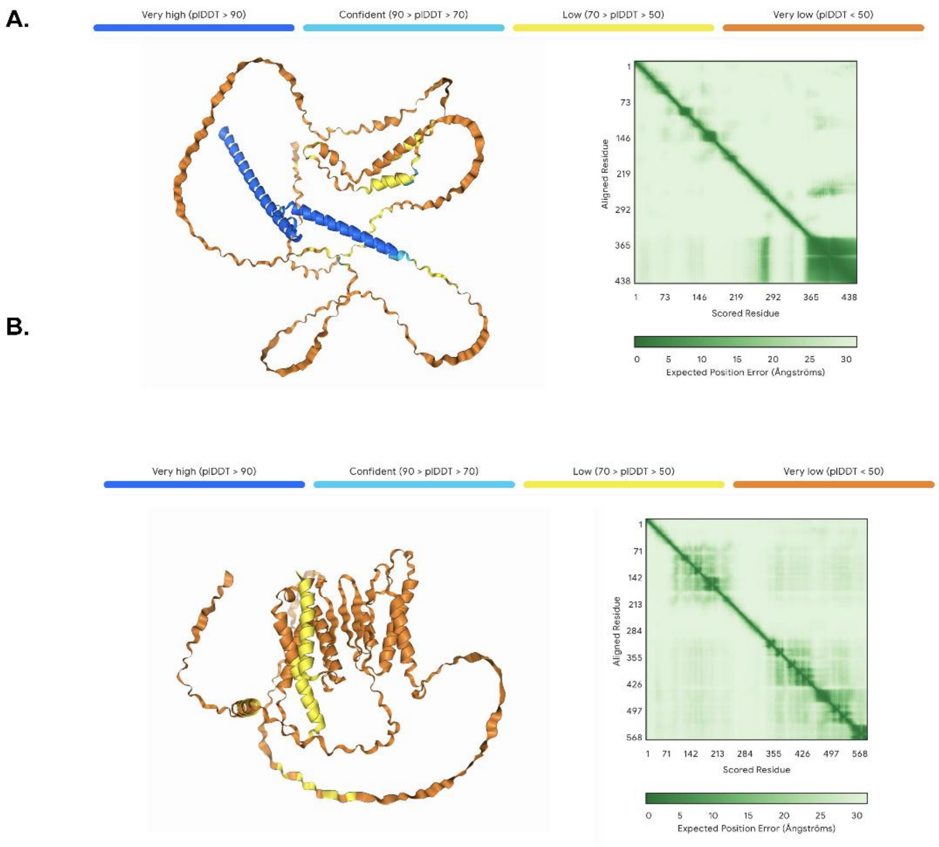

S. Figure 6. Alpha Fold prediction of crotonylated c-Myc shows a more compacted structure. (A) Prediction of unmodified c-Myc protein is shown. **(B)** Prediction of c-Myc structure after addition of 10 crotonylated residues.

S. Table 1. Mass spectrometry sequencing results of c-Myc in HEK293 cells exposed to crotonate.

Peptide	-10logP	Mass	Length	Ppm	m/z	Charge	PTM
R.C(+57.02)HVSTHQHNYAAPPSTR.K	129.05	1961.892	17	-1.2	981.9584	2	Carbamidomethylation
R.GHSVC(+57.02)STSSLYLQDLSAAASEC(+57.02)IDPSVVFPYPLNDSSSPK.S	121.2	4284.983	40	4.8	1429.351	3	Carbamidomethylation
R.SGLC(+57.02)SPSYVAVTPFSLR.G	117.47	1839.919	17	1	920.9738	2	Carbamidomethylation
K.KATAYILSVQAEEQK.L	114.45	1677.894	15	0	839.9598	2	
K.NIIIQDC(+57.02)MWSGFSAAAK.L	111.67	1910.902	17	0.4	637.9791	3	Carbamidomethylation
K.NIIIQDC(+57.02)M(+15.99)WSGFSAAAK.L	110.88	1926.897	17	0.8	964.4629	2	Carbamidomethylation; Oxidation (M)
R.SESGSPSAGGHSKPPHSPLVLK.R	110.78	2155.102	22	-0.4	719.3792	3	
R.GDNDGGGGSFSTADQLEM(+15.99)VTELLGGDM(+15.99)VNQSFIC(+57.02)DPDDETFIK.N	109.98	4612.968	43	2.9	1538.678	3	Oxidation (M); Carbamidomethylation
K.ATAYILSVQAEEQK.L	109.72	1549.799	14	0.3	1550.817	1	
R.GDNDGGGGSFSTADQLEMVTELLGGDM(+15.99)VNQSFIC(+57.02)DPDDETFIK.N	109.19	4596.973	43	4.8	1533.349	3	Oxidation (M); Carbamidomethylation
R.GDNDGGGGSFSTADQLEMVTELLGGDMVNQSFIC(+57.02)DPDDETFIK.N	108.72	4580.978	43	1.7	1146.261	4	Carbamidomethylation
K.ATAYILSVQAEEQKLISEEDLLR.K	105.7	2618.38	23	4.6	873.8105	3	
K.RSESGSPSAGGHSKPPHSPLVLK.R	104.9	2311.203	23	1.5	578.8128	4	
K.RC(+57.02)HVSTHQHNYAAPPSTR.K	98.5	2117.993	18	-1.6	707.0084	3	Carbamidomethylation
R.DQIPELENNEK.A	97.1	1327.626	11	0.7	1328.643	1	
K.KFELLPTPPLSPSR.R	95.53	1580.893	14	3.6	527.9769	3	
R.SFFALRDQIPELENNEK.A	95.3	2049.017	17	0.3	684.0176	3	
R.C(+57.02)HVSTHQHNYAAPPSTRK.D	93.14	2089.986	18	-3.1	419.0061	5	Carbamidomethylation

K.K(+68.03)ATAYILSVQAEEQK.L	88.22	1745.92	15	5.1	873.9775	2	Crotonylation
K.LVSEKLASYQAAR.K	87.77	1434.783	13	7.8	718.4092	2	
R.NYDLDYDSVQPYFYC(+57.02)DEEENFYQQQQSELQPPAPSEDIWK.K	85.56	5105.177	41	5.9	1277.318	4	Carbamidomethylation
K.SC(+57.02)ASQDSSAFSPSSDSLLSSTESSPQGSPEPLVLHEETPPTTSSDSEEEQEDEEEIDVVSVEK.R	85.06	6724.926	63	3.7	1346.007	5	Carbamidomethylation
K.FELLPTPPLSPSR.R	84.76	1452.798	13	0.8	727.4115	2	
K.KFELLPTPPLSPSRR.S	75.97	1736.994	15	1.1	435.2591	4	
K.DSGSPNPAR.G	75.92	899.4097	9	-3.1	450.7137	2	
MPLNVSFTNR.N	75.82	1177.591	10	2.9	589.8086	2	
K.KATAYILSVQAEEQKLISEEDLLR.K	75.16	2746.475	24	1.4	687.6316	4	
R.RTHNVLER.Q	73.12	1023.557	8	-1.7	342.1948	3	
R.THNVLER.Q	72.45	867.4562	7	-3.2	434.7369	2	
M.PLNVSFTNR.N	69.54	1046.551	9	2.6	524.2876	2	
R.SSDTEENVKR.R	68.31	1163.542	10	-3.8	388.8557	3	
K.LVSEK(+68.03)LASYQAAR.K	68.18	1502.809	13	0	752.4169	2	Crotonylation
R.SSDTEENVK.R	67.58	1007.441	9	-1.8	504.7301	2	
R.DQIPELENNEKAPK.V	66.74	1623.81	14	-0.5	542.2808	3	
K.LISEEDLLR.K	64.65	1086.592	9	0.5	544.3072	2	
R.SESGSPSAGGHSKPPHSPLVLKR.C	63.01	2311.203	23	0.6	386.2106	6	
K.LASYQAAR.K	61.68	878.461	8	0.3	440.2408	2	
K.LISEEDLLRK.R	58.33	1214.687	10	2	405.9065	3	
K.HKLEQLR.N	57.67	922.5348	7	-4.4	462.2758	2	
K.RSESGSPSAGGHSKPPHSPLVLKR.C	57.46	2467.304	24	-1.9	412.2266	6	
R.KDYPAAK.R	55.23	791.4177	7	-2.9	396.7177	2	
R.SESGSPSAGGHSKPPHSPLVLK(+68.03)R.C	53.58	2379.23	23	-1.5	595.8177	4	Crotonylation
R.NYDLDYDSVQPYFYC(+57.02)DEEENFYQQQQSELQPPAPSEDIWKK.F	51.95	5233.273	42	2.5	1309.337	4	Carbamidomethylation
K.VVILKKATAYILSVQAEEQK.L	49.59	2230.294	20	-3.6	744.4408	3	

R.GDNDGGGGSFSTADQLEMVTELLGGDM(+15.99)VNQSFIC(+57.02)DPDDETFIK(+68.03).N	48.54	4664.999	43	-3.5	1167.261	4	Oxidation (M); Carbamidomethylation; Crotonylation
K.HK(+68.03)LEQLR.N	48.52	990.561	7	1.6	496.2919	2	Crotonylation
R.VKLDSVR.V	47.73	815.4865	7	-0.6	408.753	2	
K.RVKLDSVR.V	47.35	971.5876	8	-2.4	324.8712	3	
R.SESGSPSAGGHSK(+68.03)PPHSPLVLK.R	47.15	2223.128	22	-5.5	556.79	4	Crotonylation
K.APK(+68.03)VVILK.K	41.44	934.6215	8	0.6	468.3215	2	Crotonylation
R.KDSGSPNPAR.G	40.41	1027.505	10	-1.6	514.7622	2	
R.K(+68.03)DYPAAK.R	38.98	859.4439	7	-3.1	430.7308	2	Crotonylation
R.EQLKHKLEQLR.N	38.33	1420.815	11	3.2	474.617	3	
R.KDYPAAK(+68.03)R.V	37.13	1015.545	8	1.5	508.784	2	Crotonylation
K.RSFFALR.D	35.23	895.5028	7	0.3	448.7618	2	
K.RVK(+68.03)LDSVR.V	32.25	1039.614	8	0	347.5475	3	Crotonylation
R.GDNDGGGGSFSTADQLEM(+15.99)VTELLGGDM(+15.99)VNQSFIC(+57.02)DPDDETFIK(+68.03).N	29.58	4680.994	43	-1.9	937.2106	5	Oxidation (M); Carbamidomethylation; Crotonylation
K.DYPAAK(+68.03)R.V	28.91	887.4501	7	1.6	444.736	2	Crotonylation
K.FELLPTPPLSPSRR.S	25.52	1608.899	14	0	537.3104	3	
K.LEQLRNSC(+57.02)A	15.29	1089.524	9	7.9	545.7771	2	Carbamidomethylation

S. Table 2. Key Resources Table

REAGENT OR RESOURCE	SOURCE	IDENTIFIER
Antibodies		
c-MYC Polyclonal antibody	Proteintech	(Cat# 10828-1-AP, RRID: AB_2148585)
Anti-Crotonyllysine pAb	PTM Bio	(Cat# PTM-501, RRID: AB_2877694)
Anti-Crotonyllysine Mouse mAb*	PTM Bio	(Cat# PTM-502, RRID: AB_2877695)
Monoclonal ANTI-FLAG® M2 antibody produced in mouse	Sigma-Aldrich	(Cat# F1804, RRID:AB_262044)
HA tag antibody	Proteintech	(Cat# 51064-2-AP, RRID:AB_11042321)
GFP (B-2)	Santa Cruz Biotechnology	(Cat# sc-9996, RRID:AB_627695)

Cyclin E1 Antibody	Cell Signaling Technology	(Cat# 20808, RRID:AB_2783554)
Ki-67 Antibody	BD Biosciences	(Cat# 550609, RRID:AB_393778)
PARP1 antibody	Proteintech	(Cat# 13371-1-AP, RRID:AB_2160459)
Pierce™ High Sensitivity Streptavidin-HRP	Thermo Scientific	(Cat# 21330, RRID: AB_3713471)
SKP2 Polyclonal antibody	Proteintech	(Cat# 15010-1-AP, RRID:AB_2187647)
GAPDH antibody	Proteintech	(Cat# 60004-1-Ig, RRID:AB_2107436)
Anti-HA IP resin	Genscript	(Cat# L00777, RRID: AB_3677368)
Anti-DYKDDDDK IP resin	Genscript	(Cat# L00425, RRID: AB_3713473)
Bacterial and virus strains		
DH5a competent cells	NEW ENGLAND BioLabs	Cat# C2987H
NEB® Stable Competent *E. coli* (High Efficiency)	NEW ENGLAND BioLabs	Cat # C3040I
Chemicals, peptides, and recombinant proteins		
MG132	MedChemExpress	Cat# HY-13259
Cycloheximide (CHX)	VWR LIFE SCIENCE	Cat# 94271-1G
Propidium Iodide 1.0 mg/mL solution in water	Invitrogen	Cat# P3566
Sodium Acetate	Sigma Aldrich	Cat# 241245
Sodium Propionate	Sigma Aldrich	Cat# P1880
Sodium Butyrate	Sigma Aldrich	Cat# B5887
Trans-Crotonic Acid	Thermo Scientific	Cat# A15765.14
Nicotinamide	Thermo Scientific	Cat# A15970.22
Trichostatin A	Selleckchem	Cat# S1045
Geneticin	Gibco	Cat# 10131-027
Critical commercial assays		
Cell Counting Kit-8	GlpBio	Cat# GK10001
Deposited data		
Raw mass spectrometry data	Supplementary information	N/A
Raw data	Supplementary information	N/A
Experimental models: Cell lines		
Human: HEK293	ATCC	CRL-1573
Human: HCT116	ATCC	CCL-247
Human: HCT116$^{p53-/-}$	Dr. Bert Vogelstein	Johns Hopkins Medical Institutes
Human: MDA-MB-231	ATCC	HTB-26
Human: H1299	ATCC	CRL-5803
Human: H1299 with HA-c-Myc-cells	This paper	N/A

Human: H1299 with HA-c-Myc-K298N-cells	This paper	N/A
Human: H1299 with HA-c-Myc-8R cells	This paper	N/A
Human: H1299 with HA-c-Myc-2R cells	This paper	N/A
Human: H1299 with HA-pcDNA.3-cells	This paper	N/A
Human: HCT116 $^{p53-/-}$ with HA-c-Myc-cells	This paper	N/A
Human: HCT116 $^{p53-/-}$ with HA-c-Myc-8R-cells	This paper	N/A
Human: HCT116 $^{p53-/-}$ with HA-c-Myc-2R-cells	This paper	N/A
Human: HCT116 $^{p53-/-}$ with HA-pcDNA.3-cells	This paper	N/A
Experimental models: Organisms/strains		
Mice: C57BL/6 nude	Jackson Laboratory	(Strain #:000819, RRID:IMSR_JAX:000819, 6 weeks, female)
Oligonucleotides		
Primers used for qRT-PCR experiments, see Table S3	This paper	N/A
Primers used for CHIP-qPCR experiments, see Table S4	This paper	N/A
Primers used for PCR mutagenesis, see Table S5	This paper	N/A
Recombinant DNA		
PCDNA3.1-Max-GFP	This paper	N/A
PCDNA3.1-SKP2	This paper	N/A
PCDNA3.1-c-Myc fragment (1-258)-HA	This paper	N/A
PCDNA3.1-c-Myc fragment (143-360)-HA	This paper	N/A
PCDNA3.1-c-Myc fragment (251-439)-HA	This paper	N/A
PCDNA3.1-c-Myc fragment (143-439)-HA	This paper	N/A
PCDNA3.1-c-Myc-HA	This paper	N/A
PCDNA3.1-c-Myc mutant (K289R)-HA	This paper	N/A
PCDNA3.1-c-Myc mutant (K298R)-HA	This paper	N/A
PCDNA3.1-c-Myc mutant (2R)-HA	This paper	N/A
PCDNA3.1-c-Myc mutant (F3R)-HA	This paper	N/A
PCDNA3.1-c-Myc mutant (R3R)-HA	This paper	N/A
PCDNA3.1-c-Myc mutant (4R)-HA	This paper	N/A
PCDNA3.1-c-Myc mutant (5R)-HA	This paper	N/A
PCDNA3.1-c-Myc mutant (8R)-HA	This paper	N/A
PCDNA3.1-c-Myc-Flag	This paper	N/A
PCDNA3.1-c-Myc mutant (8R)-Flag	This paper	N/A
PCDNA3.1-c-Myc mutant (K298N)-HA	This paper	N/A
PCDNA3.1-HA	This paper	N/A
PCDNA3.1-Ubiquitin-His	This paper	N/A
PCDNA3.1-p14ARF-Flag	This paper	N/A
PCDNA3.1-Fbw7α -Flag	This paper	N/A
Software and algorithms		

ImageJ	NIH	https://imagej.en.softonic.com/
GraphPad Prism	GraphPad Software	https://www.graphpad.com/
Bio-Rad Image Lab	Bio-Rad	N/A
Bio-Rad CFX Manager	Bio-Rad	N/A
Incucyte 2023A	Sartorius	N/A

*This antibody was used only in the production of Figure 1C.

S. Table 3. Table of primers used for qPCR experiments.

Gene	Forward	Reverse
Actin	CATGTACGTTGCTATCCAGGC	CTCCTTAATGTCACGCACGAT
L11	TGACGCGAGCAGCCAAGGTG	CCCGCACCTTTAGACCCTTCTCCA
PARP1	GTGTGGGAAGACCAAAGGAA	TTCAAGAGCTCCCATGTTCA
GADD45A	AGAGCAGAAGACCGAAAGGATG	CTTCGTACACCCCGACAGTG
EP300	TGCAGGCATGGTTCCAGTT	GCACACTGCCACGGATCATA
CCND1	GGAGCTGCTGCAAATGGA	GGAGGGCGGATTGGAAATG
DH5R	TCAGCAGAGAACTCAAGGAACC	GCCACCAACTATCCAGACCA
ATF4	TTCCTGAGCAGCGAGGTGT	AGCCTTGTCGCTGGAGAAC
HKII	CAAAGTGACAGTGGGTGTGG	GCCAGGTCCTTCACTGTCTC
ECA39	GTGGTGGGGACTTTTAAGGCTA	GATGAGCCAGGGTGCAATGA
BRCA1	CTTAGAGTGTCCCATCTGTCTGG	GCCCTTTCTTCTGGTTGAGA
p21	CTGGAACTGTTTTCTCTCGGTC	TGTATATTCAGCATTGTGGGA
p27	CCGGTGGACCACGAAGAGT	GCTCGCCTCTTCCATGTCTC
hTERT	ATGCGACAGTTCGTGGCTCA	ATCCCCTGGCACTGGACGTA
ITAG6	TTTGGAGCCCCGGGTACTTA	AGCAGGAACAGGAACGAGAC
FOXO3A	TGTTGGTTTGAACGTGGGGA	TGTCCACTTGCTGAGAGCAG
eIF2α	CACACACATACCTCAGAATGCC	CAAGCTGACATAAGCCCCCATT
CCNE1	GGCCAAAATCGACAGGAC	GGGTCTGCACAGACTGCAT
E2F1	TCCAAGAACCACATCCAGT	CTGGGTCAACCCCTCAAG
CDC25A	AGGGTCTGGGCAGTGATTATG	CAGCTTCTGAGGTAGGGAATGT
LDHA	ACGTGCATTCCCGATTCCT	CATGCCAACAGCACCAACC
CAD	GTTCGGTCTCTGCAAGTGGT	AACTTCTTGGTCAGCTCCCG

S. Table 4. Table of primers used for CHIP-qPCR experiments.

Promoter	Forward	Reverse
E2F2	TCACCCCTCTGCCATTAAAGG	AGCAGTGTATTCCCCAGGCC
Nucleolin	TTGCGACGCGTACGAGCTGG	ACTCCGACTAGGGCCGATAC
eIF4F	AAGCCTCTCGTTACTCACGC	AGATTCAAACCGATTGGCC
HKI	CATTGGACCAGGGAGACAGT	GGTGTGGACACTGGAGGTTT
CYP1A2	GAGGCTTAGGCAGGAGGATT	AACAACTGAGGCACGCTATG

S. Table 5. Table of primers used for PCR mutagenesis.

Primer	Forward	Reverse
K392R & K398R	GCCCCCAGGGTAGTTATCCTTAAAAGAGCCACAGCAT	CTACCCTGGGGGCCTTTTCATTGTTTTCCAACTCCGG
K430R	GCGGAAACGACGAGAACAGTTGAAACACAGACTTGAAC	TCGTCGTTTCCGCAACAAGTCCTCTTCAGAAATGAGC
K148R	TGGCCTCCTACCAGGCTGCGCGCAAAGACAGCGG	CCTGGTAGGAGGCCAGCCTCTCTGAGACGAGCTTGG
K298R & K289R	CACAGCCCACTGGTCCTCAGGAGGTGCCACGT	ACCAGTGGGCTGTGAGGAGGTCTGCTGTGGCC
K326& K317	CAAGAGGGTCAGGTTGGACAGTGTCAGAGTCCTGAGAC	CAACCTGACCCTCTTGGCAGCAGGATAGTCCCTCC
K298N	CTCAACAGGTGCCACGTCTCCACACATCAGCACAACTACG	CGTGGCACCTGTTGAGGACCAGTGGGCTGTGAGGAGG
K289R	GAGGCCACAGCAGACCTCCTCACAG	CTGTGAGGAGGTCTGCTGTGGCCTC
K298R	CACTGGTCCTCAGGAGGTGCCACGTC	GACGTGGCACCTCCTGAGGACCAGTG
K275R	CAGGCTCCTGGCAGAAGGTCAGAGTCTG	CAGACTCTGACCTTCTGCCAGGAGCCTG
K317R	CCCTCCACTCGGAGGGACTATCCTGCTG	CAGCAGGATAGTCCCTCCGAGTGGAGGG
K323R	CTATCCTGCTGCCAGGAGGGTCAAGTTGG	CCAACTTGACCCTCCTGGCAGCAGGATAG
K326R	CCAAGAGGGTCAGGTTGGACAGTGTC	GACACTGTCCAACCTGACCCTCTTGG
