# Supplementary material for: Crotonylation impedes c-Myc oncogenic activity
Source: Proc Natl Acad Sci U S A. 2026 Jun 1;123(23):e2530020123. doi: 10.1073/pnas.2530020123 (PMC13250510; doi:10.1073/pnas.2530020123)

Figure 1A

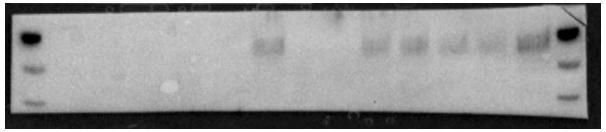

IB:pan-cro

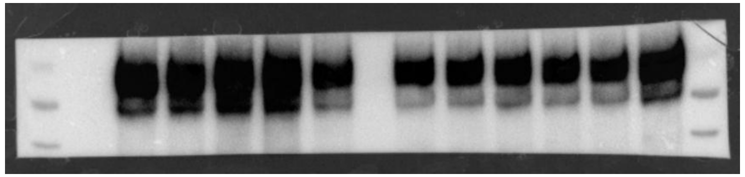

IB:c-Myc

Figure 1B

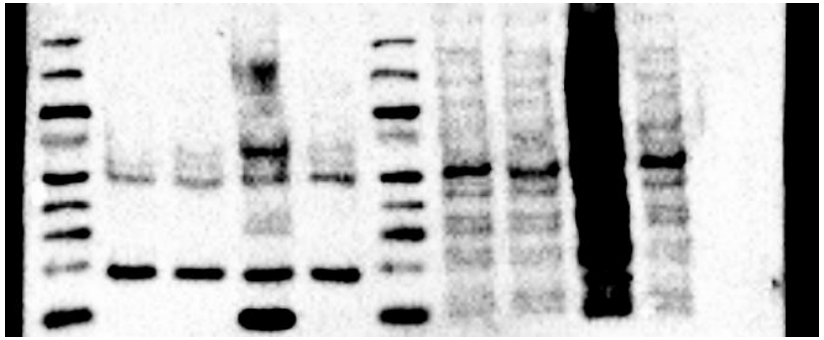

IB:pan-cro

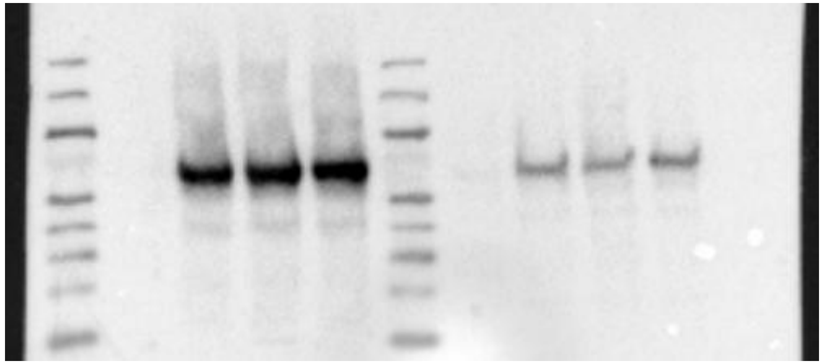

IB:c-Myc

Figure 1C

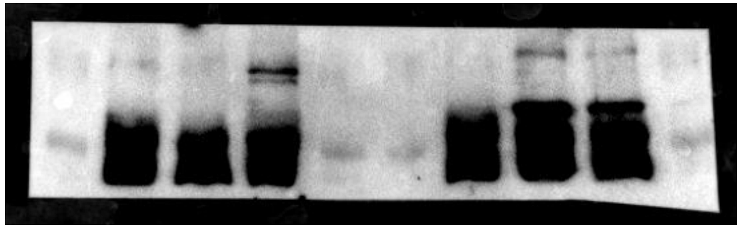

IB:pan-cro

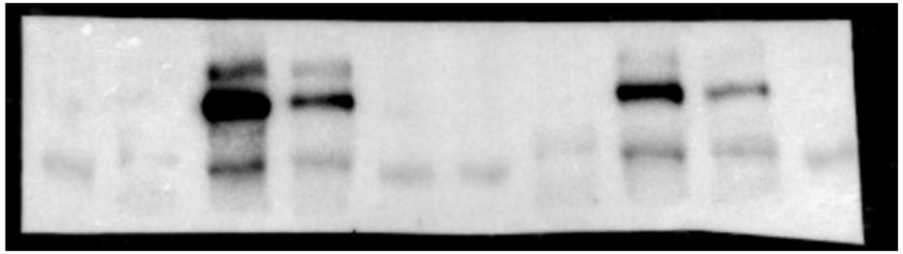

IB :c-Myc

Figure 1D

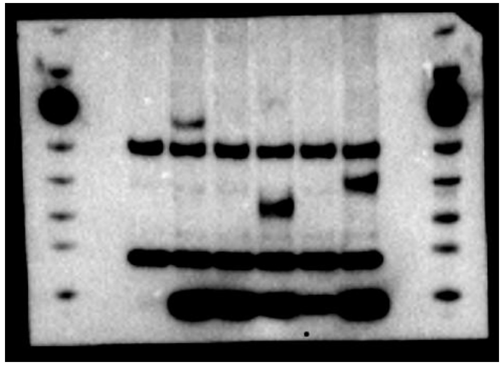

IB:pan-cro

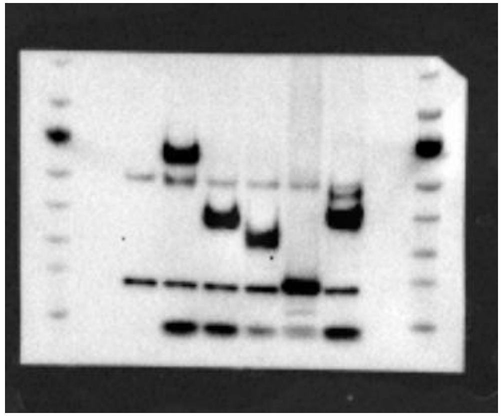

IB:HA

Figure 1E

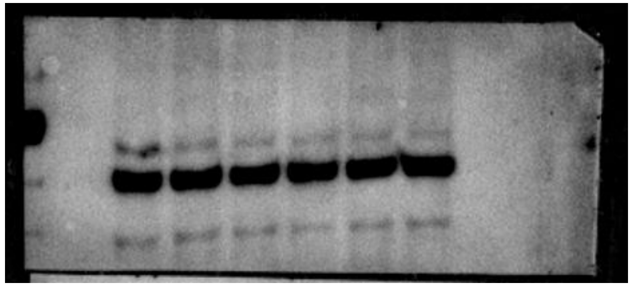

IB:pan-cro

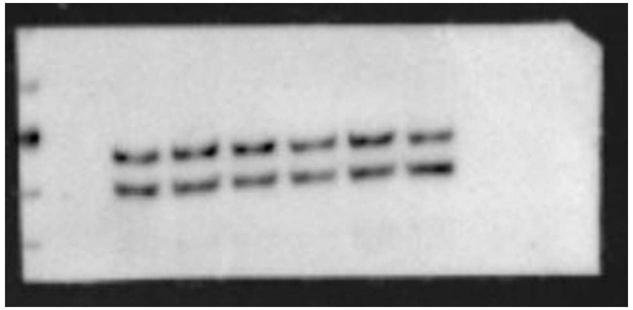

IB:HA

Figure 1F

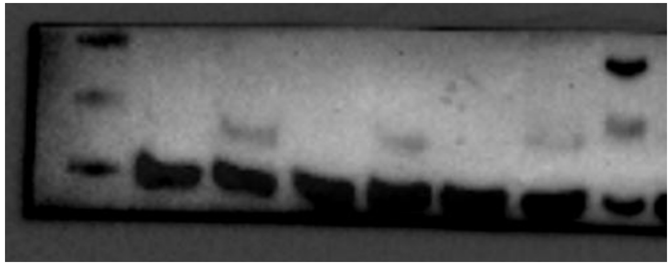

IB:pan-cro

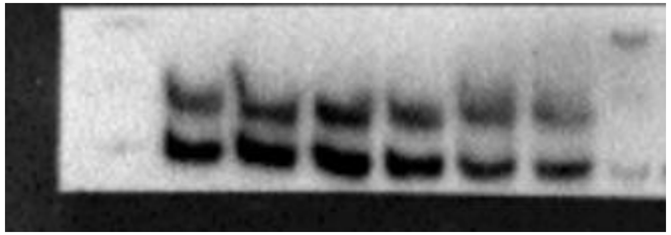

IB:c-Myc

Supp Figure 1B

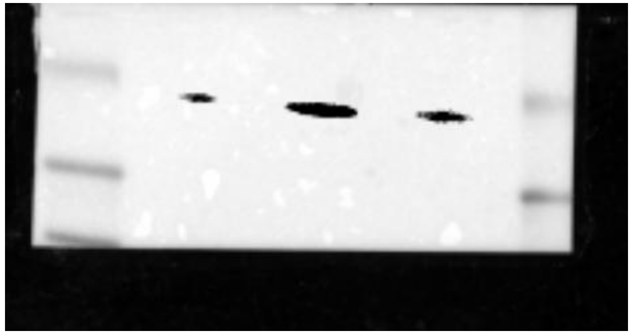

IB: Streptavidin

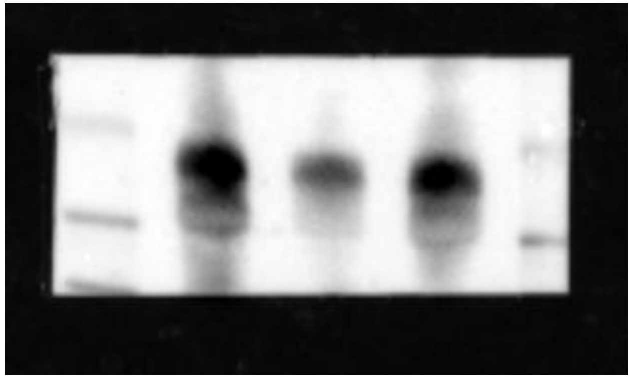

IB: Myc

Figure 2D

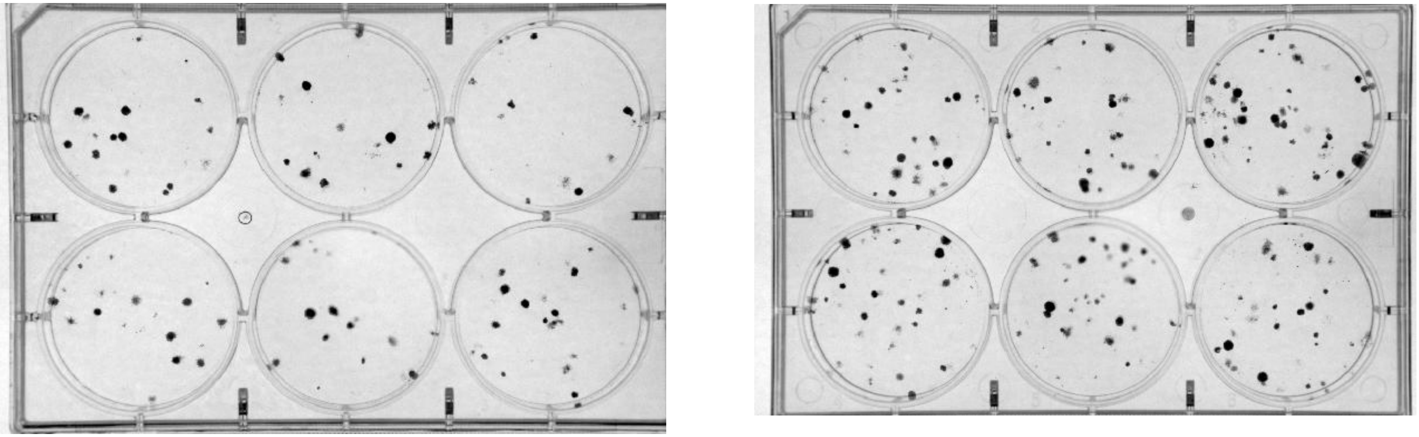

WT

8R

Figure 2E

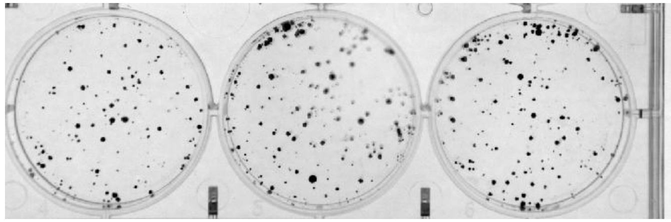

WT

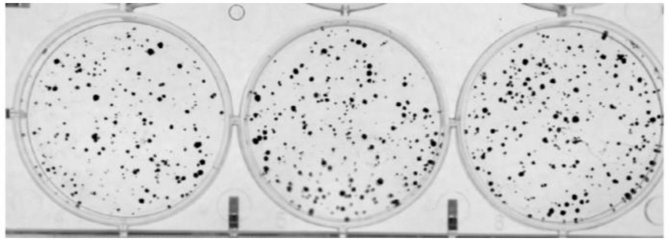

8R

Figure 2F

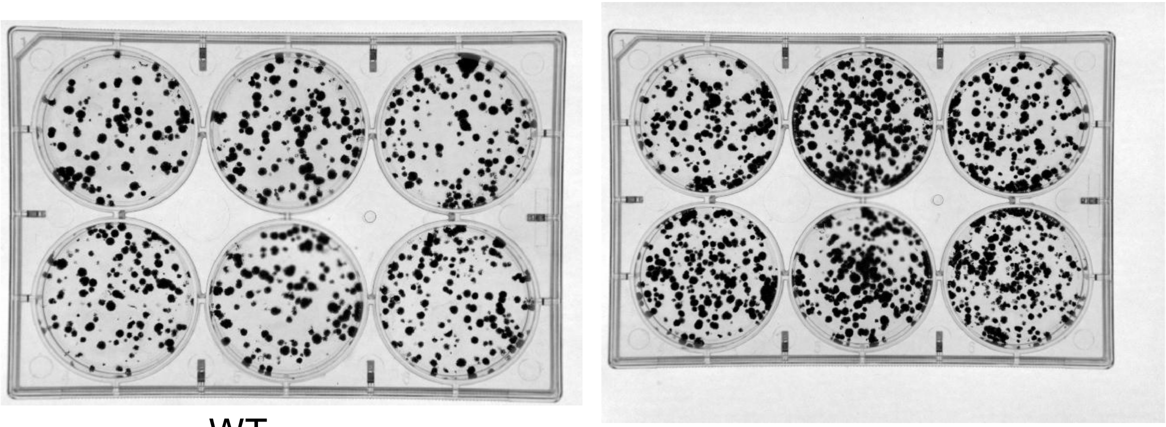

WT

2R

Figure 3E

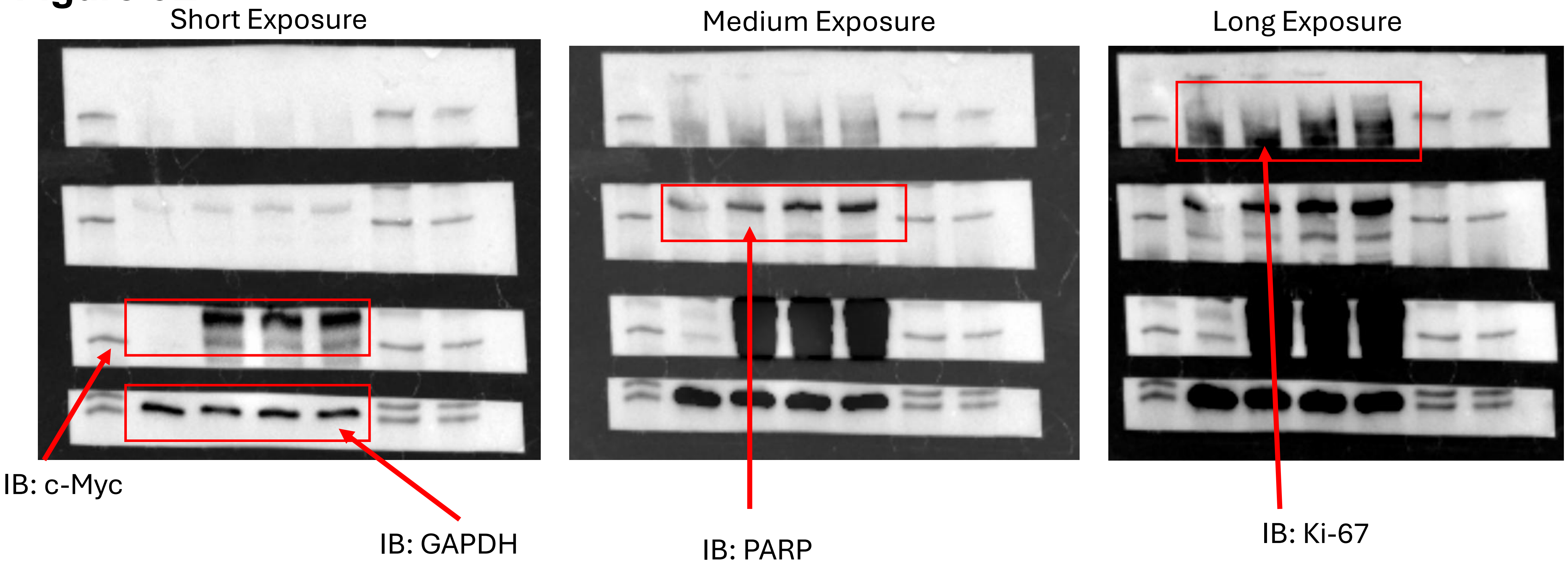

Short Exposure
Medium Exposure
Long Exposure
IB: c-Myc
IB: GAPDH
IB: PARP
IB: Ki-67

Supplemental 3A

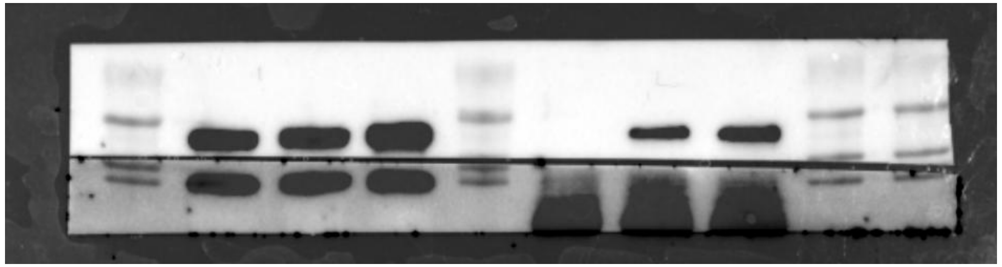

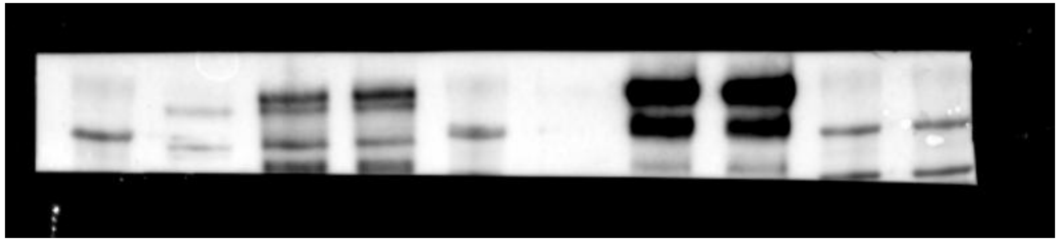

Figure 4A

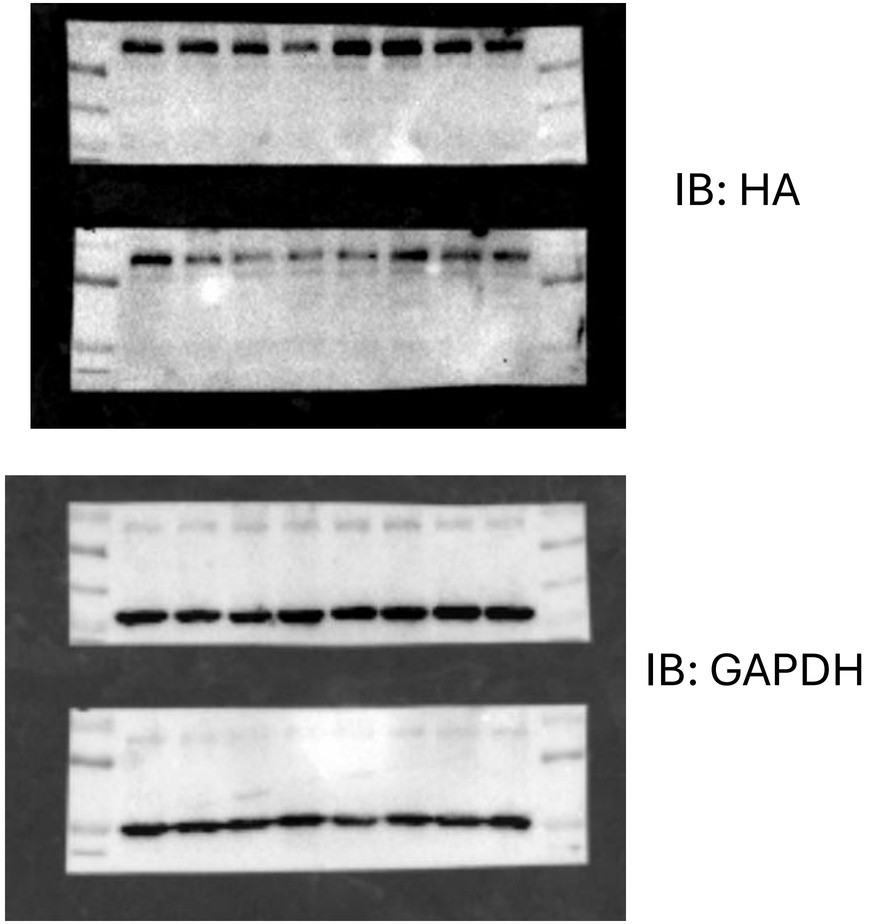

Figure 4B

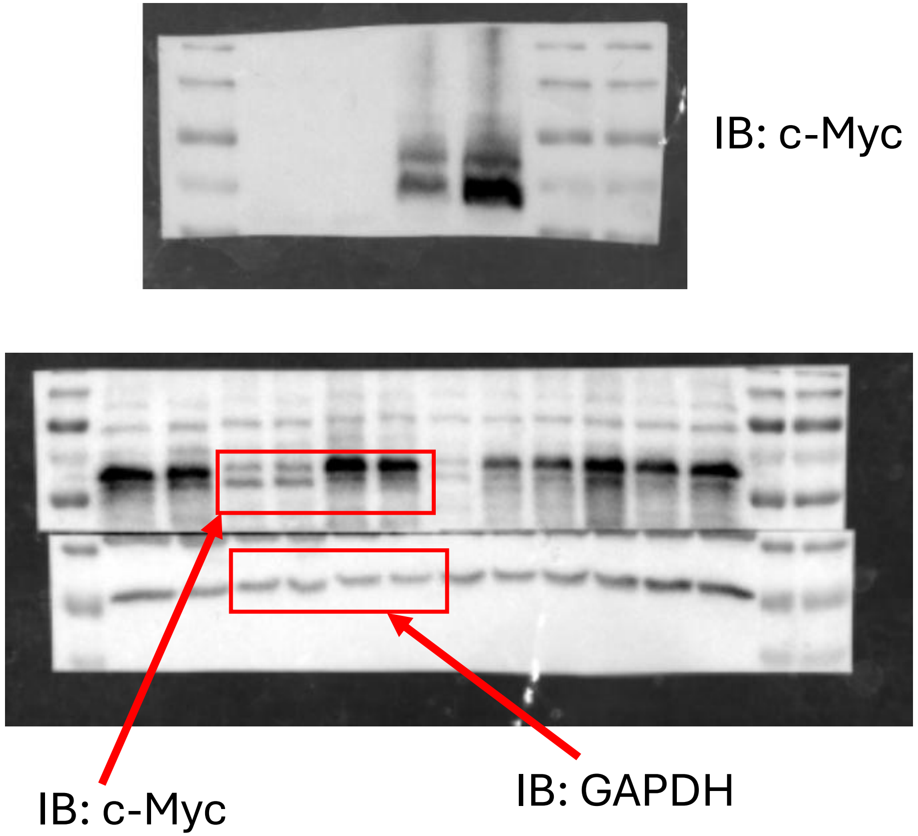

Figure 4C

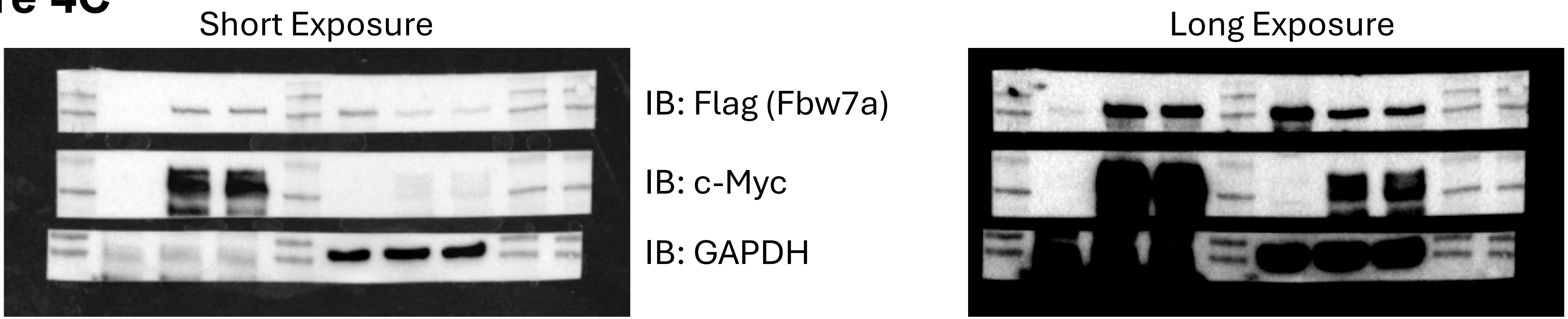

Figure 4D

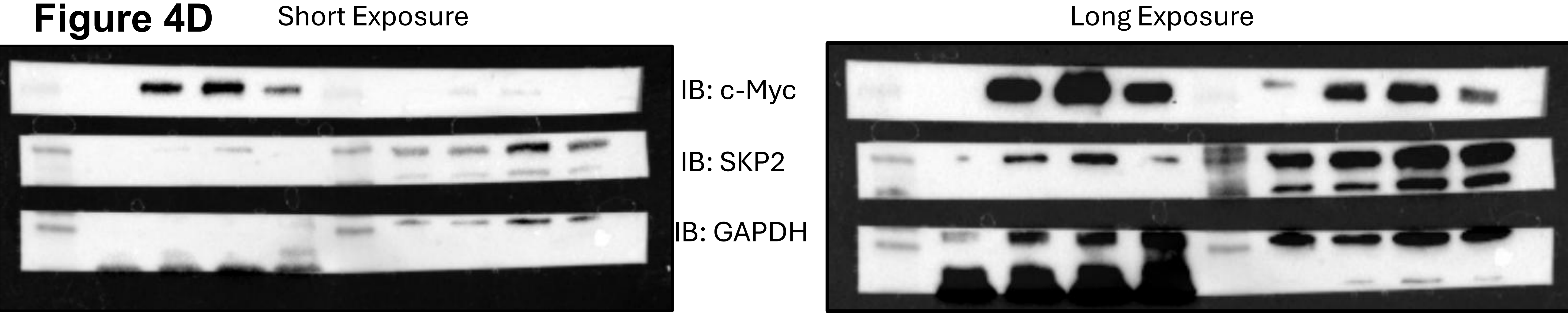

Figure 4E

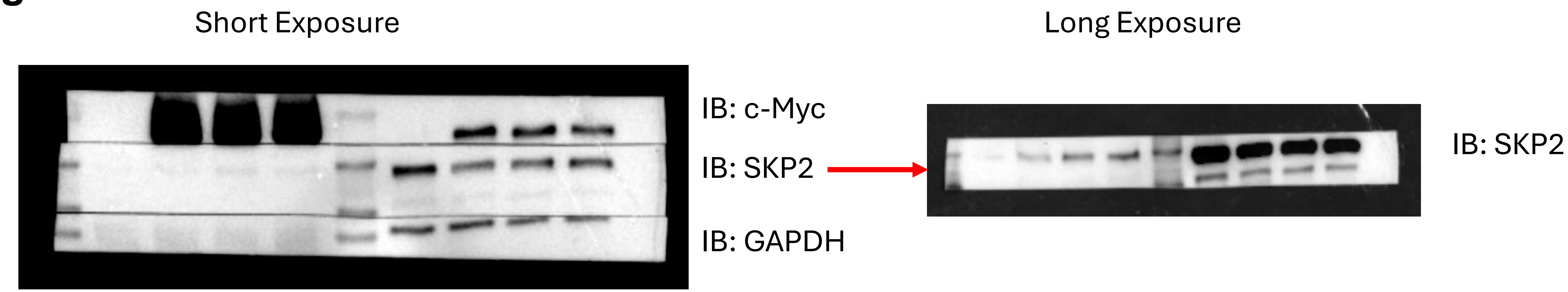

Figure 4F

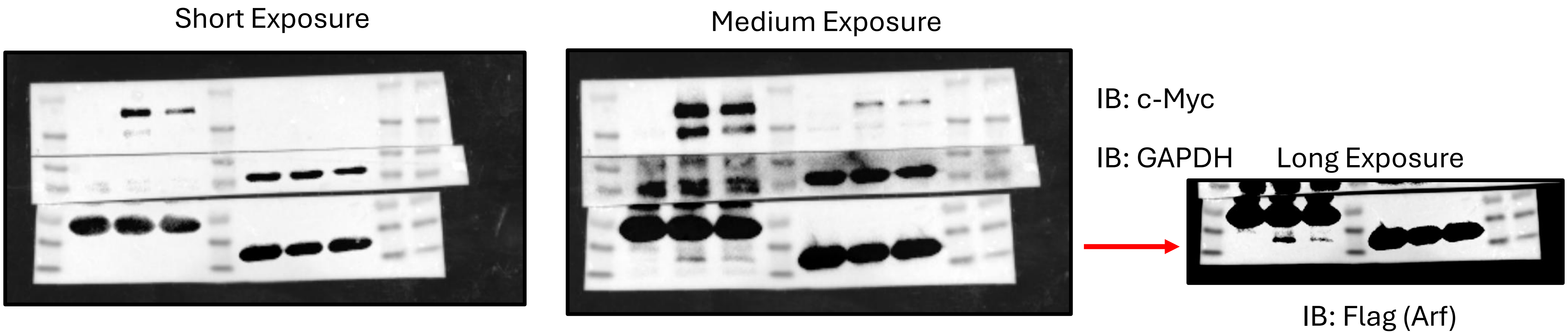

Figure 4G

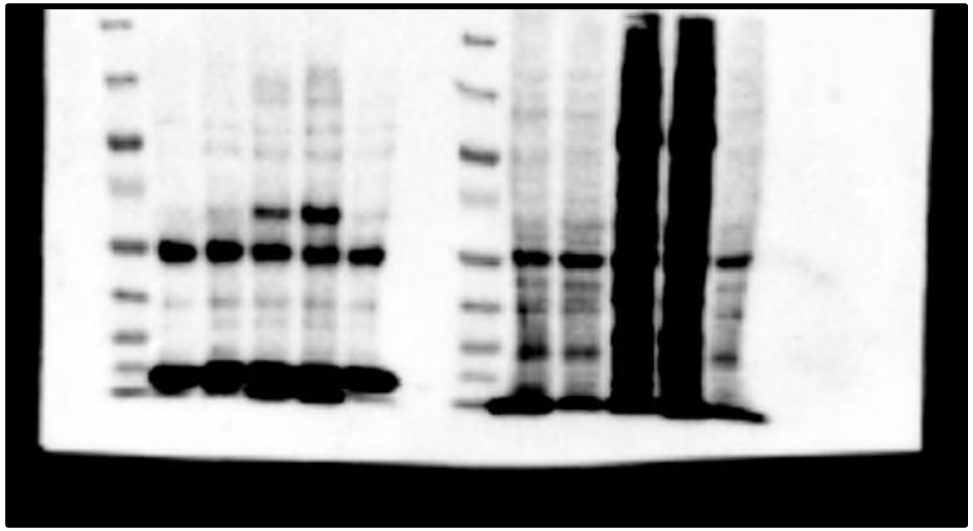

IB:pan-cro

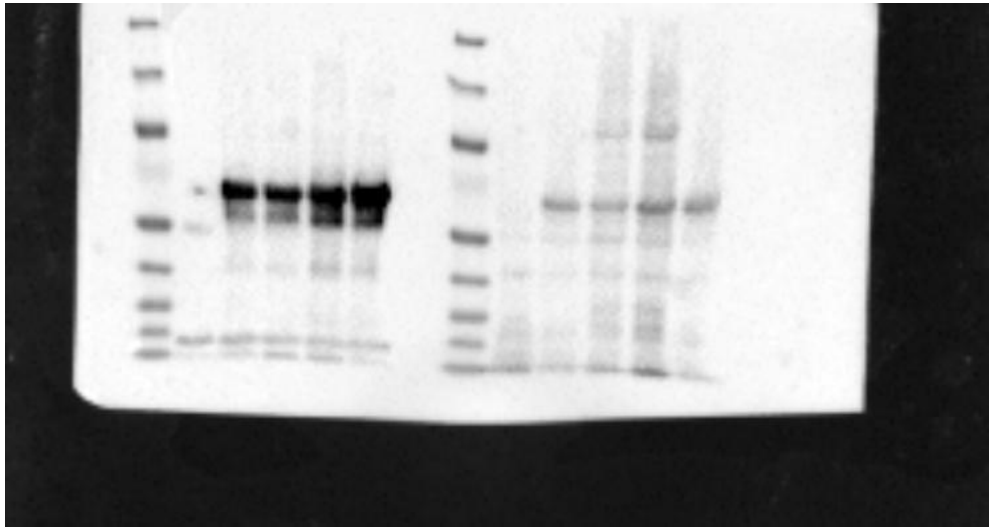

IB:c-Myc

Figure 4H

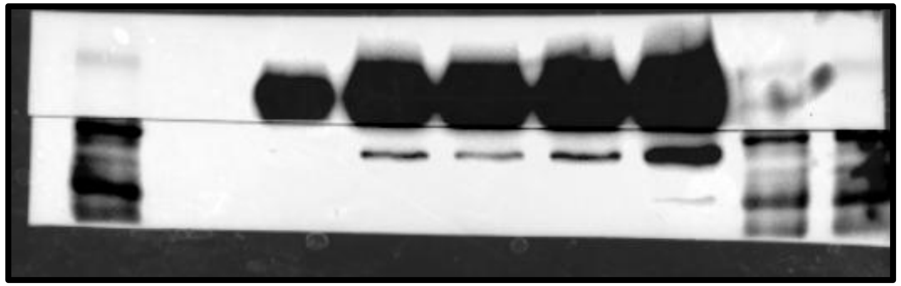

IB:c-Myc

IB:SKP2

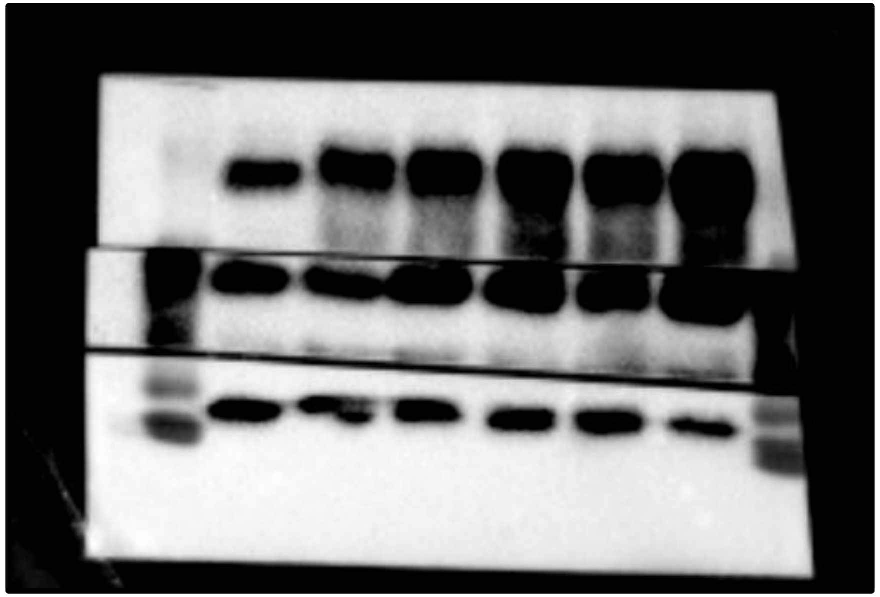

IB: c-Myc

IB: SKP2

IB: GAPDH

Supplemental 4B

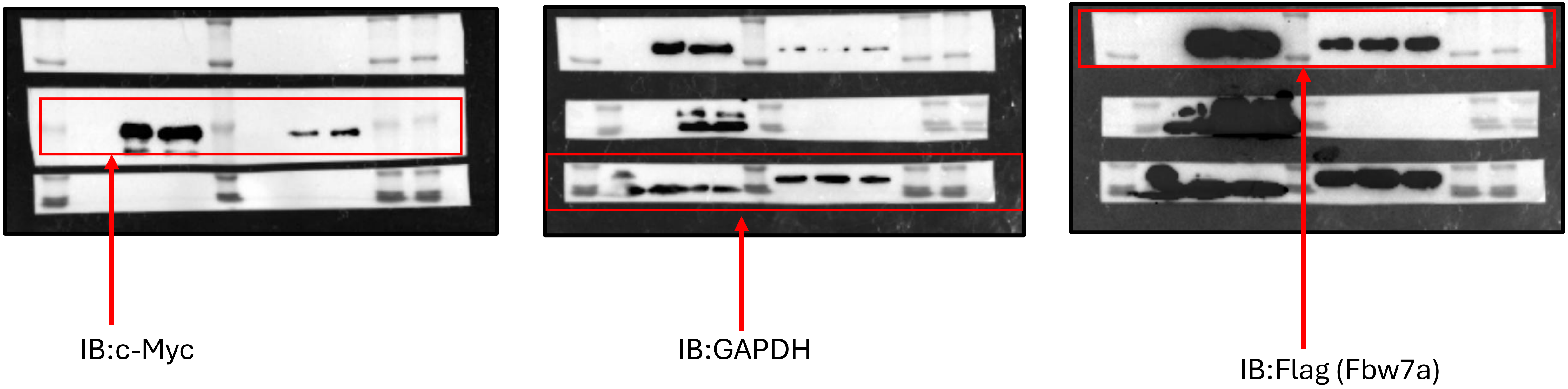

Figure 5A

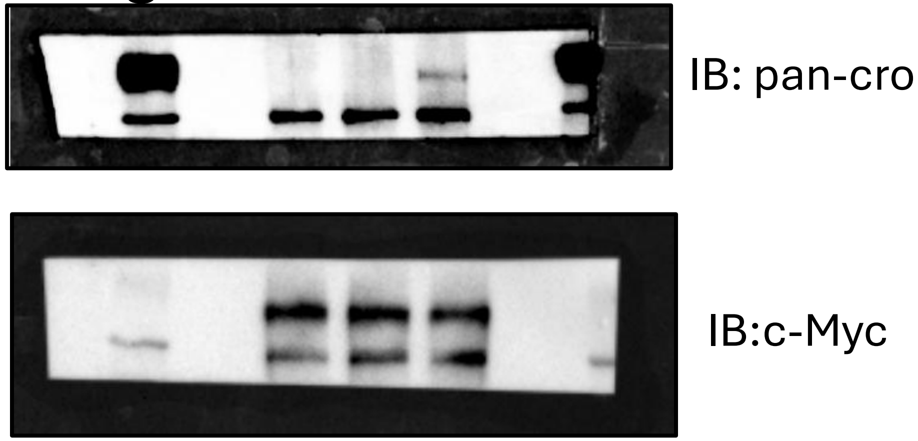

Supplemental 5A

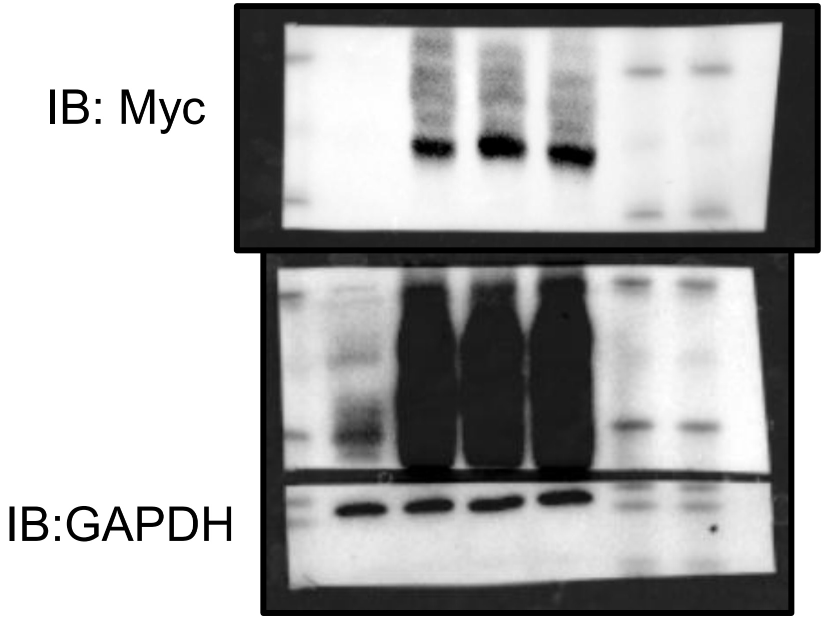

Figure 5C

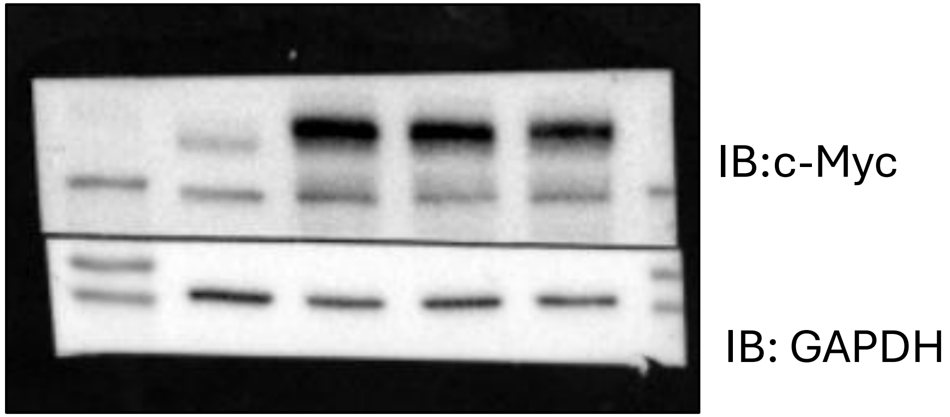

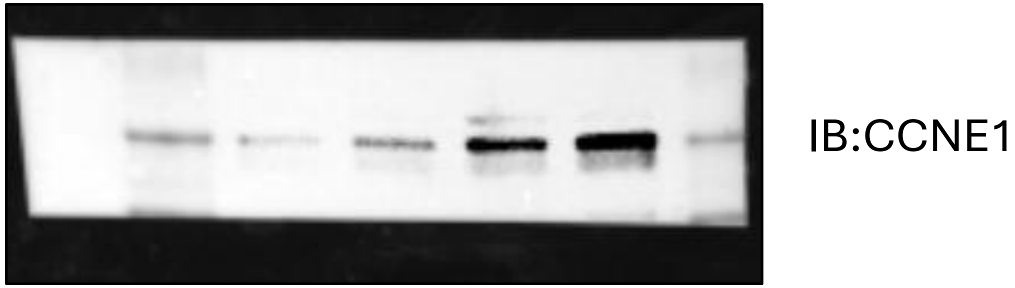

Figure 5D

WT

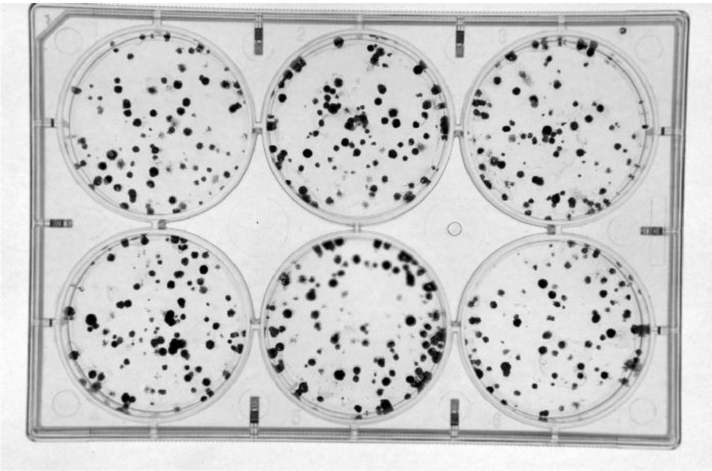

WT

K298N

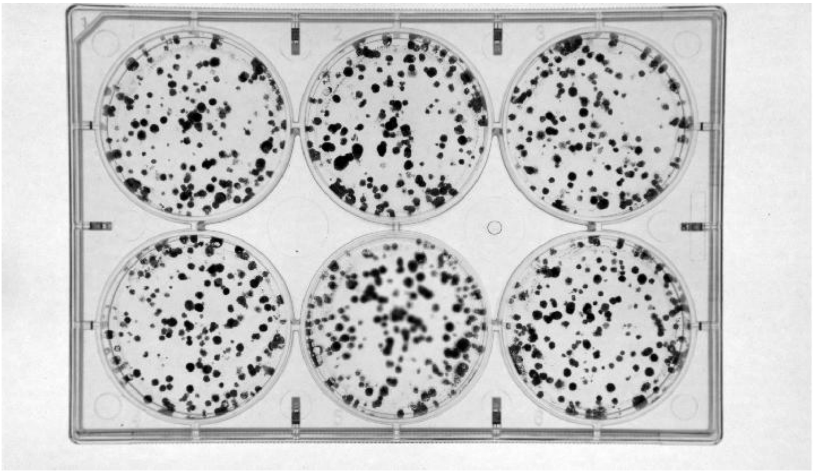

K298N

8R

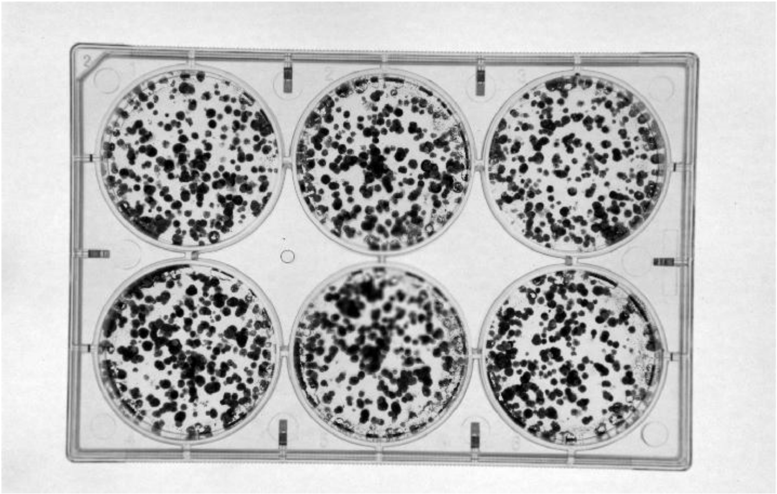

8R

Supplemental 5B

IB: HA (top membrane)

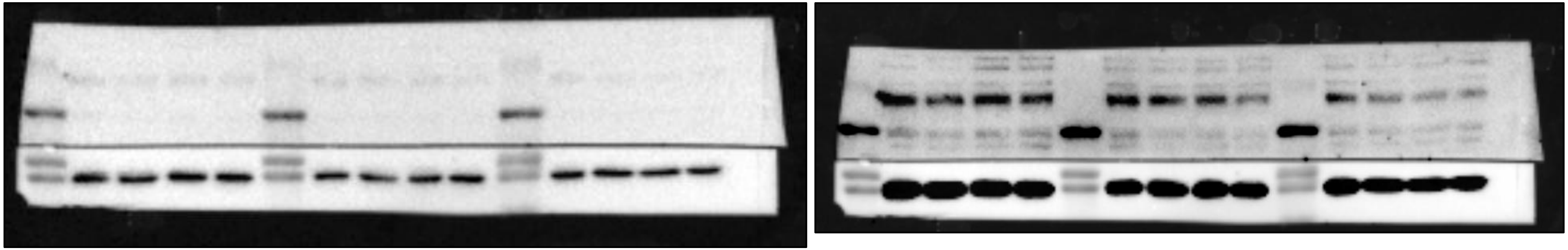

IB: GAPDH (bottom membrane)

Figure 6A

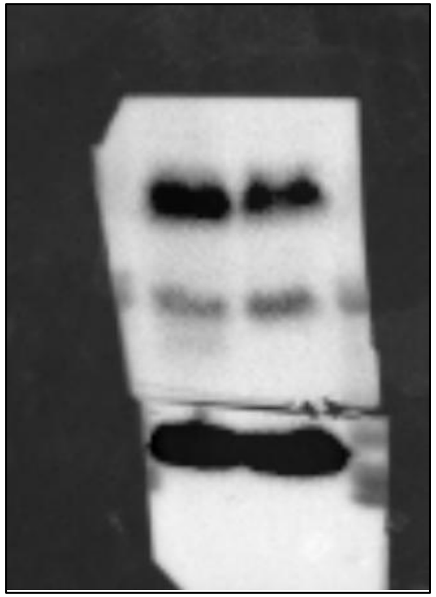

IB:c-Myc

IB: GAPDH

Figure 6E

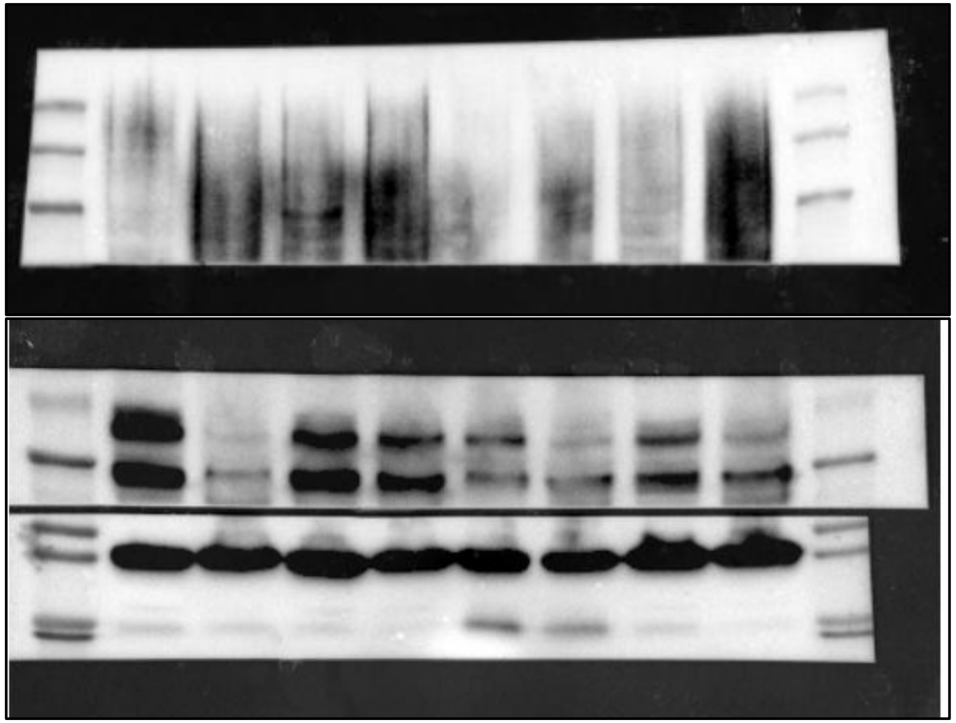

Figure 6F

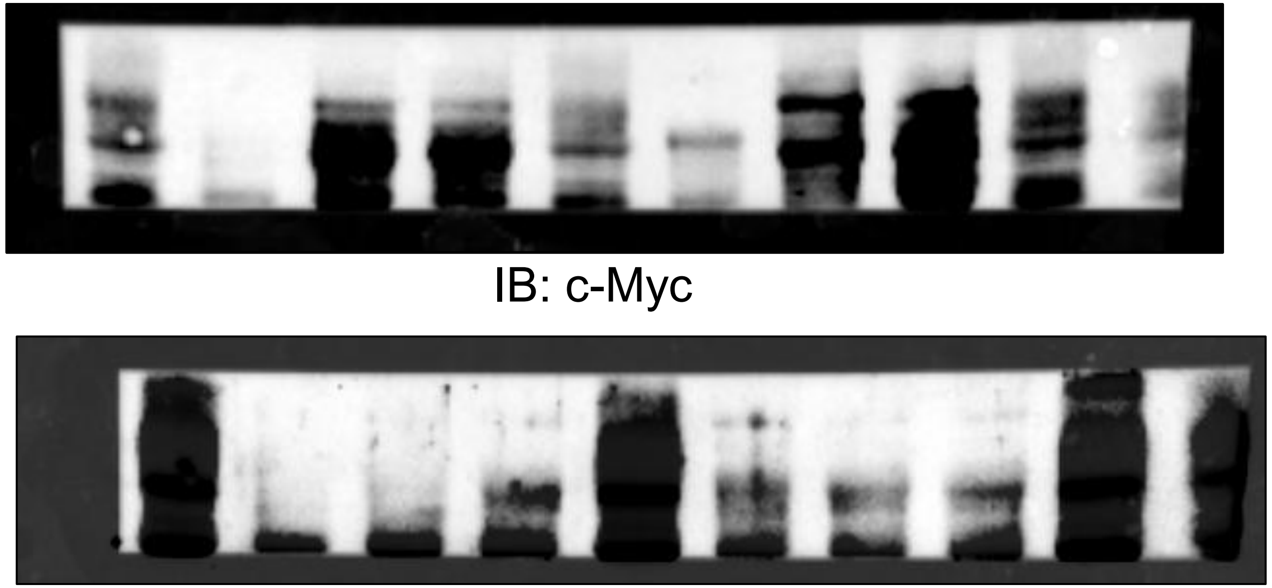

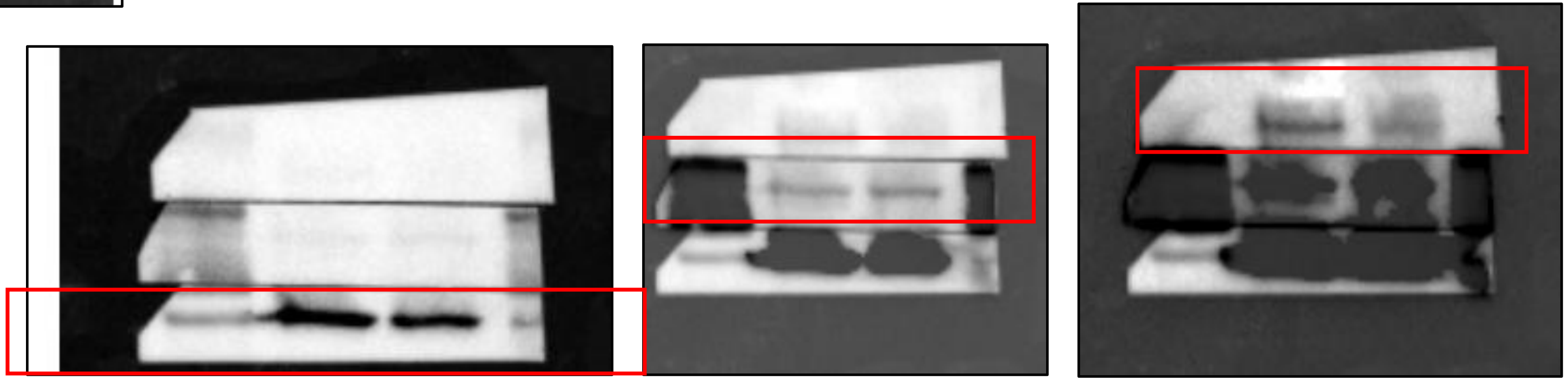

Supplement: Supplementary file 2 — Dataset S01 (PDF) [file pnas.2530020123.sd01.pdf]
